# Evolution of the Twist Subfamily Vertebrate Proteins: Discovery of a Signature Motif and Origin of the Twist1 Glycine-Rich Motifs in the Amino-Terminus Disordered Domain

**DOI:** 10.1371/journal.pone.0161029

**Published:** 2016-08-24

**Authors:** Yacidzohara Rodriguez, Ricardo R. Gonzalez-Mendez, Carmen L. Cadilla

**Affiliations:** 1 Department of Biochemistry, School of Medicine, University of Puerto Rico, San Juan, Puerto Rico, United States of America; 2 Department of Radiological Sciences, School of Medicine, University of Puerto Rico, San Juan, Puerto Rico, United States of America; University of South Florida, UNITED STATES

## Abstract

Twist proteins belong to the basic helix-loop-helix (bHLH) family of multifunctional transcriptional factors. These factors are known to use domains other than the common bHLH in protein-protein interactions. There has been much work characterizing the bHLH domain and the C-terminus in protein-protein interactions but despite a few attempts more focus is needed at the N-terminus. Since the region of highest diversity in Twist proteins is the N-terminus, we analyzed the conservation of this region in different vertebrate Twist proteins and study the sequence differences between Twist1 and Twist2 with emphasis on the glycine-rich regions found in Twist1. We found a highly conserved sequence motif in all Twist1 (SSSPVSPADDSLSNSEEE) and Twist2 (SSSPVSPVDSLGTSEEE) mammalian species with unknown function. Through sequence comparison we demonstrate that the Twist protein family ancestor was “Twist2-like” and the two glycine-rich regions found in Twist1 sequences were acquired late in evolution, apparently not at the same time. The second glycine-rich region started developing first in the fish vertebrate group, while the first glycine region arose afterwards within the reptiles. Disordered domain and secondary structure predictions showed that the amino acid sequence and disorder feature found at the N-terminus is highly evolutionary conserved and could be a functional site that interacts with other proteins. Detailed examination of the glycine-rich regions in the N-terminus of Twist1 demonstrate that the first region is completely aliphatic while the second region contains some polar residues that could be subject to post-translational modification. Phylogenetic and sequence space analysis showed that the Twist1 subfamily is the result of a gene duplication during Twist2 vertebrate fish evolution, and has undergone more evolutionary drift than Twist2. We identified a new signature motif that is characteristic of each Twist paralog and identified important residues within this motif that can be used to distinguish between these two paralogs, which will help reduce Twist1 and Twist2 sequence annotation errors in public databases.

## Introduction

Class B tissue restricted basic helix-loop-helix (bHLH) Twist proteins are transcription factors expressed in different tissues during early stages of embryogenesis and their presence is essential for proper development and survival [1–3]. In mammals, Twist1 and Twist2 paralogs are known for playing a major role in the inhibition of differentiation of mesenchymal cell lineages, particularly in bone, muscle and dermis [4–7] The maintenance of functional Twist proteins throughout evolution has been of great importance since mutations in *Twist* genes and improper regulation of genes that are targeted by these transcription factors can result in human diseases such as Setleis Syndrome [8], Barber-Say Syndrome and Ablepharon Macrostomia [9], Saethre Chotzen syndrome [10,11], inflammatory diseases [12] and cancer [13,14].

It is well documented that Twist proteins are characterized by at least three distinct motifs: (i) the basic region which is necessary for DNA binding, (ii) the HLH domain needed for protein dimerization, (iii) and a Twist box domain which can function as either an activation or repression domain [1,15]. Over the years, an interesting paradigm in the literature has emerged in which the bHLH protein dimer, as well as other factors such as DNA binding to E box (5’-CANNTG-3’) sequences, influences the repression or activation roles of these factors. In general, the availability of other bHLH proteins within the cell influences dimer choice [16–18]. Frequently, inhibitory complexes between dimers occur through the HLH domain; however, emerging information in the literature shows that these factors are not only able to form multi-protein repressor complexes but also to bind and block the transactivation activity of other transcription factors through their C-terminal and N-terminal regions [19–22]. The role of the C-terminus in interactions has been well established as in the case of MyoD and MEF2 during muscle differentiation [21,23,24], RunX2 during regulation of the osteoblastic program [19] and p53 during cancer development [25]. However, few studies have been reported regarding the role of the N-terminus in protein-protein interactions: pCAF and p300 during chromatin remodeling [22,24] and PGC-1α during brown fat thermogenesis [26]. There are other studies on the other hand, as in the case of ADD1/SREBP1c during energy homeostasis involving adipose tissue [27], in which the domain used by Twist2 to interact with ADD1/SREBP1c remains yet to be determined but the HLH region is excluded.

Although there have been attempts to characterize the roles of the N-terminal, bHLH, and C-terminal structural domains of Twist proteins, the bHLH and C-terminal domains have received most of the attention. However, based on the interactions reported involving the amino terminus, it is of great importance to focus on this region as well. Since the major difference between Twist1 and Twist2 is found in their N-terminus region, where Twist1 contains two glycine-rich motifs not present in Twist2, the main purpose of this study is to compare the sequence differences located in the amino-terminus region of vertebrate Twist1 and Twist2 proteins. We focused on questions that remain to be elucidated with regards to these regions. For example, were these glycine-rich regions first present in the original ancestral *Twist* gene or were they acquired/lost later throughout evolution? What class of vertebrates was the first to develop these regions and which glycine-rich region was developed first? What is the function of these glycine-rich regions in protein-protein interactions? Could such sequence differences be used to further explain or differentiate their individual modes of action? In addition, even though the major differences between these two proteins are found in the amino terminus, the first 50 amino acids of Twist1 and Twist2 are almost identical and highly conserved. Therefore, could this region contain any new conserved sequences/structural motifs that could be used to further explain their interactions with other proteins?

When searching in public databases for sequences belonging to Twist family members of transcription factors, these can be differentiated from other transcription factors by an amino acid identity of 89% or higher in the bHLH region. Twist subfamily of proteins can be differentiated from the other members of the Twist family by the highly conserved Twist box domain that comprises the last 20 amino acids of the carboxy terminus. Normally, in order to discriminate between Twist1 and Twist2 sequences, one looks for the presence or absence of the glycine-rich regions, since these are only present in Twist1 sequences. However, this is sometimes not enough and has presented a problem because it has led to incorrect annotation of Twist sequences. Here, we focus on the amino terminus region of vertebrate Twist1 and Twist2 proteins and determine the origin and evolution of human Twist1 glycine-rich motifs. We also review recent data that suggests a possible role for these glycine-rich motifs and suggest a way to reduce Twist1 and Twist2 errors in sequence annotation in public databases by demonstrating the existence of a new Twist protein signature motif in the amino terminus region that is characteristic for each member of the Twist protein subfamily.

Throughout evolution, *Twist* genes have been conserved from jellyfish to human [1,28]. It has been suggested that among the vertebrate *Twist* genes only one protochordate *Twist* gene underwent more than one gene duplication event before the teleost-tetrapod split [28]. This event gave origin to three ancestral *Twist* genes. The sequences of such duplicated *Twist* genes underwent further modifications such as deletions and/or duplications, which gave rise to the different Twist1, Twist2 and Twist3 paralogs now present in various vertebrate species [28,29]. The *Branchiostoma belcheri* (Lancelet) Twist protein has previously been detected as a true *Twist* gene [28] and it has been considered as closer to the ancestor of all vertebrate Twist proteins [1]. In this paper we have analyzed 68 protein Twist sequences that span 5 vertebrate classes to study the molecular evolution of the Twist subfamily.

## Results

### The ancestor Twist protein was “Twist2-like”

**Fig 1:**
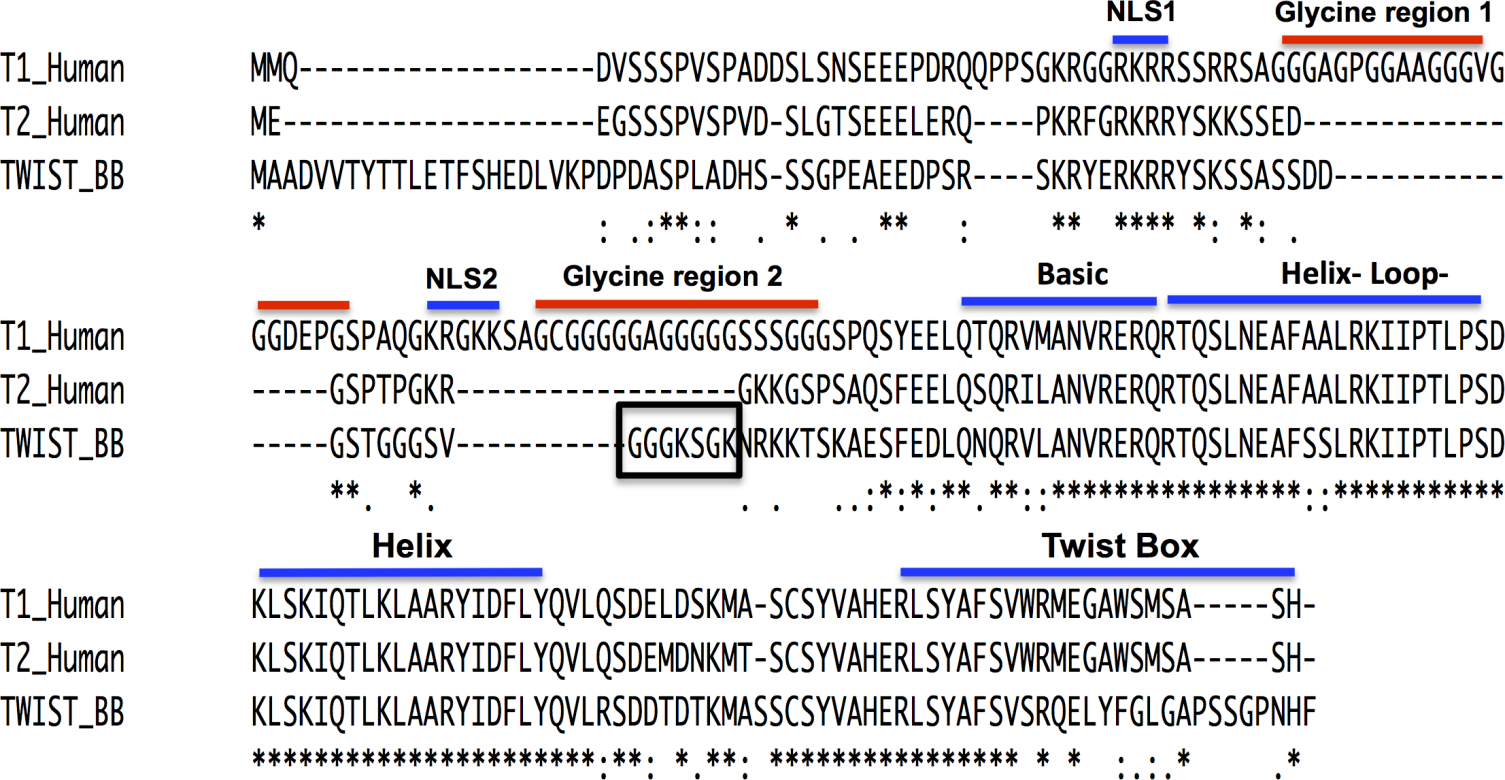
Amino acid comparison between ancestor and human Twist sequences. Functional motifs (nuclear localization signal (NLSs), basic Helix-loop-Helix (bHLH), glycine-rich regions, and Twist box domains) are depicted by bars on top of the sequence. Twist2 lacks both glycine regions present only in Twist1. The Twist_BB (closest to ancestor) protein lacks the first glycine region but contains some glycine residues in the second glycine region (black box). The Twist_BB protein contains extra amino acid residues at the start of its N-terminus and in the Twist box domain of the C-terminus not present in either human Twist proteins. The bHLH domains are approximately 90% conserved in all three sequences. Overall, Twist1 amino acid sequence is 54% similar to Twist_BB, while Twist2 has 64% similarity, which suggests that the ancestor of both Twist paralogs was a “Twist2-like” protein. Below the protein sequence: (*) = conserved residues; (:) = conservative mutations; (.) = semi-conservative mutations; () = non-conservative mutations. Twist_BB = *Branchiostoma belcheri* (Lancelet). Sequence names used represent the common name of the species to which they belong. The alignment was performed with PSI-COFFEE.

In order to examine whether these glycine-rich regions were first present in the original ancestral *Twist* gene we compared the sequence of human (*Homo sapiens*) Twist1 and Twist2 proteins with an extant protein that is thought to resemble the ancestral Twist protein sequence (Twist_BB), from the Lancelet (*Branchiostoma belcheri*); a cephalochordate that is considered to be closest to the ancestor of all vertebrate *Twist* genes [1] (Tables 1 and 2, Fig 1). The basic helix-loop-helix (bHLH) domains of human and the Twist_BB proteins are approximately 90% conserved. The Twist_BB protein contains amino acids that are not present in either of the human Twist sequences, particularly at the beginning of the N-terminus and in the Twist box domain of the C-terminus. It can be inferred from this finding that these regions were not needed or important in the function of the protein and therefore were deleted or lost throughout evolution. As already described, Twist1 contains two glycine-rich regions that are not present in Twist2. Interestingly, the Twist_BB protein lacks the first region; however, it has four glycine residues in the second region as depicted by the black box. We can speculate that after duplication of the Twist_BB gene, the few glycine residues that were originally present in the second glycine region underwent further mutation and duplication events throughout evolution that made it gain the two glycine-rich regions now present in human Twist1, as described [1]. For example, a mechanism of simple tandem trinucleotide repeats could generate one glycine-rich region and a duplication/recombination event can generate the other glycine-rich region. In general, when compared to the Twist_BB protein, the amino acid sequence of human TWIST1 shares 54% amino acid identity while TWIST2 shares 64%, respectively (Fig 1). Taking these findings into account, we suggest that the ancestral Twist sequence was a “Twist2-like” protein.

**Table 1:** Taxa set for MSA and phylogenetic analyses for Twist1 sequences.

Vertebrate Group	Accession Number	Sequence Name	Species	Common Name
Mammal	[NCBI-Protein:NP_000465]	T1_Human	*Homo sapiens*	Human
Mammal	[NCBI-Protein:NP_001178074.1]	T1_Cow	*Bos taurus*	Bovine
Mammal	[NCBI-Protein:XP_011729410.1]	T1_PgTlMac	*Macaca nemestrina*	Pig-tailed Macaque
Mammal	[Swiss-Prot:Q8MIH1]	T1_Marmost	*Callithrix jacchus*	White-tufted-ear marmoset
Mammal	[Swiss-Prot:Q8MI03]	T1_Chimp	*Pan troglodytes*	Chimpanzee
Mammal	[NCBI-Protein:NP_035788.1]	T1_Mouse	*Mus musculus*	Mouse
Mammal	[NCBI-Protein:NP_445982.1]	T1_Rat	*Rattus norvegicus*	Rat
Mammal	[NCBI-Protein:XP_011992033.1]	T1_WildShp	*Ovis orientalis musimon*	Wild Sheep
Mammal	[NCBI-Protein:XP_006085118.1]	T1_LilBat	*Myotis lucifugus*	*Little brown bat
Mammal	[NCBI-Protein:XP_008146579.1]	T1_BigBat	*Eptesicus fuscus*	*Big Brown Bat
Mammal	[NCBI-Protein:XP_011923490.1]	T1_MngMkey	*Cercocebus atys*	Sooty Mangabey Monkey
Mammal	[NCBI-Protein:XP_003130240.2]	T1_Pig	*Sus scrofa*	Pig
Mammal	[NCBI-Protein:XP_862829.1]	T1_Dog	*Canis lupus familiaris*	Dog
Mammal	[NCBI-Protein:XP_002818224.1]	T1_Orangut	*Pongo abelii*	Sumatran orangutan
Mammal	[NCBI-Protein:XP_004839639.1]	T1_MoleRat	*Heterocephalus glaber*	Naked mole-rat
Mammal	[NCBI-Protein:XP_004415621.1]	T1_Walrus	*Odobenus rosmarus divergens*	Walrus
Mammal	[NCBI-Protein:NP_001139637.1]	T1_Horse	*Equus caballus*	Horse
Mammal	[NCBI-Protein:XP_007455457.1]	T1_Dolphin	*Lipotes vexillifer*	Yangtze River dolphin
Mammal	[NCBI-Protein:XP_004263506.1]	T1_KlWhale	*Orcinus orca*	Killer Whale
Mammal	[NCBI-Protein:XP_004468012.1]	T1_Armdllo	*Dasypus novemcinctus*	Nine-banded Armadillo
Mammal	[NCBI-Protein:XP_005343917]	T1_Vole	*Microtus ochrogaster*	Prairie Vole (Rodent)
Bird	[NCBI-Protein:NP_990070]	T1_Chicken	*Gallus gallus*	Chicken
Bird	[NCBI-Protein:XP_002190104.1]	T1_Finch	*Taeniopygia guttata*	Zebra Finch
Bird	[NCBI-Protein:XP_005518918.1]	T1_GrndTit	*Pseudopodoces humilis*	Tibetan ground-tit
Fish	[NCBI-Protein:NP_571059.1]	T1_Zfish	*Danio rerio*	Zebra Fish
Fish	[NCBI-Protein:NP_001098069.1]	T1_FugFish	*Takifugu rubripes*	Fugu Japanese Fish
Fish	[NCBI-Protein:NP_001098177.1]	T1_MdkFish	*Oryzias latipes*	Japanese rice fish (Medaka)
Fish	[NCBI-Protein:XP_010738912.1]	T1_YCroakr	*Larimichthys crocea*	Yellow croaker
Fish	[NCBI-Protein:XP_007260751.1]	T1_CavFish	*Astyanax mexicanus*	Mexican Tetra Blind Cave Fish
Fish	[NCBI-Protein:DAA06073.1]	T1_StkleBk	*Gasterosteus aculeatus*	Three-spined Stickleback
Fish	[NCBI-Protein:DAA06078.1]	T1_Puffer	*Tetraodon nigroviridis*	Spotted Green Pufferfish
Reptile	[NCBI-Protein:NP_001280041.1]	T1_Lizard	*Anolis carolinensis*	Lizard
Reptile	[NCBI-Protein:XP_005303213.1]	T1_PtTrtle	*Chrysemys picta bellii*	Western Painted Turtle
Amphibian	[NCBI-Protein:NP_001091211.1]	T1_AfFrog	*Xenopus laevis*	African Clawed Frog
Amphibian	[NCBI-Protein:NP_989415.1]	T1_WeFrog	*Xenopus (Silurana) tropicalis*	Western Clawed Frog

A total of 35 Twist1 protein sequences were identified based on sequence homology to Human Twist1 sequence in five major vertebrate classes through the NCBI Protein Database and the UniProtKB database using the BLAST algorithm.

*Additional Twist1 sequences added only for the phylogenetic analysis.

**Table 2:** Taxa set for MSA and phylogenetic analyses for Twist2 and Ancestor sequences.

**Vertebrate group**	**Accession Number**	**Sequence Name**	**Species**	**Common Name**
Mammal	[NCBI-Protein:NP_476527]	T2_Human	*Homo sapiens*	Human
Mammal	[NCBI-Protein:NP_067723]	T2_Rat	*Rattus norvegicus*	Rat
Mammal	[NCBI-Protein:XP_003309597.1]	T2_Chimp	*Pan troglodytes*	Chimpanzee
Mammal	[NCBI-Protein:XP_006083049]	T2_LilBat	*Myotis lucifugus*	*Little Brown Bat
Mammal	[NCBI-Protein:XP_008136676.1]	T2_BigBat	*Eptesicus fuscus*	*Big Brown Bat
Mammal	[NCBI-Protein:XP_002750013.1]	T2_Marmost	*Callithrix jacchus*	White-tufted-ear marmoset
Mammal	[NCBI-Protein:XP_011726175.1]	T2_PgTlMac	*Macaca nemestrina*	Pig-tailed Macaque
Mammal	[NCBI-Protein:XP_003133824.1]	T2_Pig	*Sus scrofa*	Pig
Mammal	[NCBI-Protein:XP_543311.1]	T2_Dog	*Canis familiaris*	Dog
Mammal	[NCBI-Protein:NP_001077217]	T2_Cow	*Bos taurus*	Bovine
Mammal	[NCBI-Protein:XP_011989863.1]	T2_WildShp	*Ovis orientalis musimon*	Wild Sheep
Mammal	[NCBI-Protein:NP_031881.1]	T2_Mouse	*Mus musculus*	Mouse
Mammal	[NCBI-Protein:XP_009235351.1]	T2_Orangut	*Pongo abelii*	Sumatran Orangutan
Mammal	[NCBI-Protein:XP_004868472.1]	T2_MoleRat	*Heterocephalus glaber*	Naked mole-rat
Mammal	[NCBI-Protein:XP_011898211.1]	T2_MngMkey	*Cercocebus atys*	Sooty Mangabey Monkey
Mammal	[NCBI-Protein:XP_004410484.1]	T2_Walrus	*Odobenus rosmarus divergens*	Walrus
Mammal	[NCBI-Protein:XP_008526060.1]	T2_Horse	*Equus przewalskii*	Wild Horse
Mammal	[NCBI-Protein:XP_007470002.1]	T2_Dolphin	*Lipotes vexillifer*	Yangtze River dolphin
Mammal	[NCBI-Protein:XP_004262587.1]	T2_KlWhale	*Orcinus orca*	Killer Whale
Mammal	[NCBI-Protein:XP_004455664.1]	T2_Armdllo	*Dasypus novemcinctus*	Nine-banded Armadillo
Mammal	[NCBI-Protein:XP_005361737.1]	T2_Vole	*Microtus ochrogaster*	Prairie Vole (Rodent)
Bird	[NCBI-Protein:NP_990010]	T2_Chicken	*Gallus gallus*	Chicken
Bird	[NCBI-Protein:XP_002190783.1]	T2_Finch	*Taeniopygia guttata*	Zebra Finch
Bird	[NCBI-Protein:XP_005532751]	T2_GrndTit	*Pseudopodoces humilis*	Tibetan ground-tit
Fish	[NCBI-Protein:NP_001005956.1]	T2_Zfish	*Danio rerio*	Zebra Fish
Fish	[NCBI-Protein:NP_001098070.1]	T2_FugFish	*Takifugu rubripes*	Fugu Japanese Fish
Fish	[NCBI-Protein:DAA06067.1]	T2_MdkFish	*Oryzias latipes*	Japanese rice fish (Medaka)
Fish	[NCBI-Protein:XP_010738189.1]	T2_YCroakr	*Larimichthys crocea*	Yellow croaker
Fish	[NCBI-Protein:XP_007256435.1]	T2_CavFish	*Astyanax mexicanus*	Mexican Tetra Blind Cave Fish
Fish	[NCBI-Protein:DAA06075.1]	T2_StkleBk	*Gasterosteus aculeatus*	Three-spined Stickleback
Fish	[NCBI-Protein:DAA06080.1]	T2_Puffer	*Tetraodon nigroviridis*	Spotted Green Pufferfish
Reptile	[NCBI-Protein:XP_003215189.1]	T2_Lizard	*Anolis carolinensis*	Lizard
Reptile	[NCBI-Protein:XP_005299959.1]	T2_PtTrtle	*Chrysemys picta bellii*	Western Painted Turtle
**Protochordate group**	**Accession Number**	**Sequence Name**	**Species**	**Common Name**
Cephalochordate	[Swiss-Prot:O96642.1]	Twist_BB	*Branchiostoma belcheri*	Belcher's lancelet
Cephalochordate	[NCBI-Protein:XP_002606170.1]	Twist_BF	*Branchiostoma floridae*	Florida lancelet
Cephalochordate	[NCBI-Protein:AFJ79493.1]	I2DBA7_BRALA	*Branchiostoma* l*anceolatum*	Common lancelet
Cephalochordate	[NCBI-Protein:AFJ79489.1]	I2DBA3_BRALA	*Branchiostoma* l*anceolatum*	Common lancelet
Tunicate	[UniProtKB:F7B554]	F7B554_CIOIN	*Ciona intestinalis*	*Yellow sea squirt*
Tunicate	[UniProtKB:Q75UU2]	Q75UU2_CIOSA	*Ciona savignyi*	Solitary sea squirt

A total of 33 Twist2 protein sequences were identified based on sequence homology to the Human Twist2 sequence in four major representative vertebrate classes through the NCBI Protein Database and the UniProtKB database using the BLAST algorithm. A Twist protein from *Branchiostoma belcheri* (Lancelet), a member of the Protochordates, was included as the Twist protein closest to the ancestral Twist sequence for MSA analysis and as the out-group for phylogenetic analysis.

*Additional Twist2 sequences added only for the phylogenetic analysis.

It is important to note that when searching for the ancestor *twist-like* protein sequence, Twist protein sequences were searched for within the protochordates: amphioxus, C. *intestinalis* and C. *savignyi*. However, the amphioxus (lancelet) cephalochordates was chosen because as already described, it is considered the ancestor of all vertebrates [1] and shares over 89% amino acid identity across the bHLH while the others share only 45–51% bHLH region identity, in agreement with previous studies [28]. Within the amphioxus species, bHLH proteins (with 54–63% bHLH region identity) were detected in our database searches, such as proteins from the *Branchiostoma lanceolatum* species; however, these were not included in the analysis because they did not contain the conserved *twist*-specific carboxy sequence. Only two amphioxus species in our database searches against the human TWIST proteins harbored true Twist proteins and had the conserved carboxy terminal sequence: *Branchiostoma belcheri* (BB) and *Branchiostoma floridae*; both sharing 92% bHLH identity. Even though the Twist protein sequence of the *B*. *floridae* species is more similar to human Twist protein sequences and its C-terminus is more conserved than the C-terminus of the *B*. *belcheri* species, we chose the *B*. *belcheri* species as the ancestral twist-like protein because it has been reviewed and categorized as a twist gene by the Swiss-Prot database, while the *B*. *floridae* species has yet to be categorized as such. Furthermore, when the entire protein sequence of human Twist2 is compared with the *B*. *floridae* species protein using BLASTP, it has 68% amino acid identity while Twist1 has 56%. This further suggests that the ancestral Twist sequence was more of a “Twist2-like” sequence, as is also suggested when compared to the *B*. *belcheri* species, which showed 64% amino acid identity to human Twist2 and 54% identity to human Twist1, when aligned using BLASTP (S1 Fig.).

### The glycine-rich regions of Twist1 were acquired later in evolution

**Fig 2:**
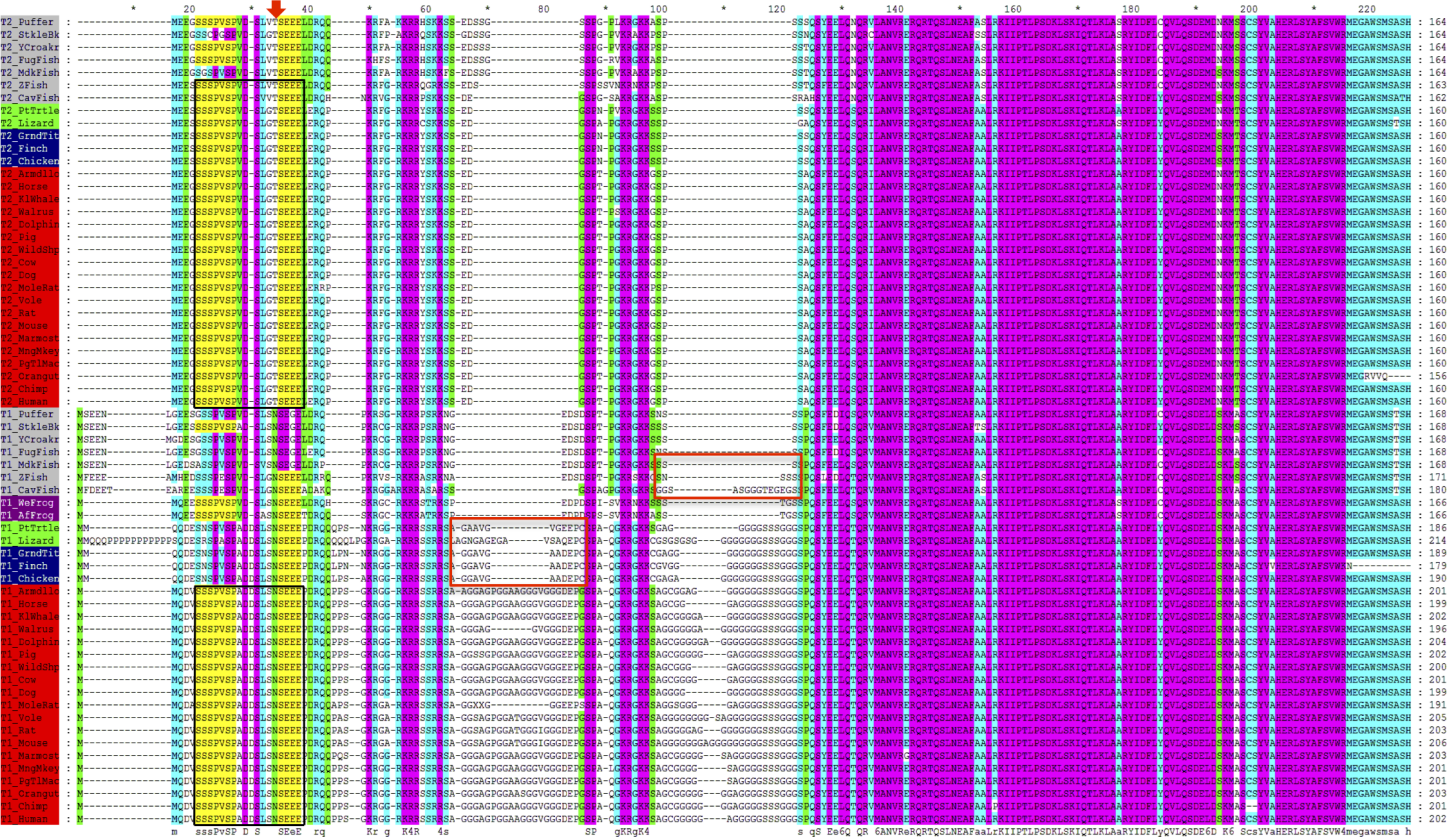
Multiple sequence alignment of Twist1 and Twist2 vertebrate protein sequences. Acquisition of glycine residues is first seen in fish (T1_Cavfish), particularly in the second glycine region (right red box), while acquisition of glycine residues in the first glycine-rich region is first seen amongst the reptiles (left red box). Twist1 amphibians lack both glycine-rich regions, as observed with all Twist2 proteins. Sequence comparison of Twist proteins in different vertebrate species demonstrates a conserved sequence found in the majority of Twist1 and Twist2 proteins, particularly amongst mammals: **SSSPVSP**ADDSLSN**SEEE** (the motif sequence for Twist1 in mammals) or **SSSPVSP**VDSLGT**SEEE** (the motif sequence for Twist2), depicted by black rectangles. Bold residues represent conserved sub-motifs that are 100% conserved within the mammalian class (highlighted in yellow in the MSA). A red arrow on top of the alignment depicts important residues (underlined threonine for Twist2 sequences and asparagine for Twist1) that are key in differentiating between Twist1 and Twist2 sequences. The alignment was colored based on the different levels of amino acid conservation: Pink represents 100% amino acid conservation, cyan blue represents 75% and green represents 50% conservation respectively. Each of the Twist1 and Twist2 proteins are grouped (highlighted) based on the vertebrate class to which they belong to: fish (grey), reptiles (green), amphibians (purple), birds (blue) and mammals (red). Gaps are indicated as hyphens. Sequence names used represent the common name of the species to which they belong. MSA was performed with T-COFFEE.

In order to determine when these glycine-rich regions were first acquired during evolution, we decided to look into the evolution of the glycine-rich motifs within the different vertebrate species. Specifically, we wanted to determine which species first started acquiring these regions. Did acquisition of both regions occur at the same time or was one developed first, followed by the other? Furthermore, did Twist2 proteins possess these regions at some point and lost them later on? In order to shed light on some of these questions, we compared Twist1 and Twist2 sequences within vertebrates by performing a multiple sequence alignment (MSA) (Fig 2). Based on sequence homology a total of 64 Twist protein sequences (Tables 1 and 2), 33 for Twist1 and 31 for Twist2, were identified in five representative vertebrate classes (mammals, avian, reptiles, amphibians and fish) by sequence database searches using the Basic Local Alignment Search Tool (BLAST) [30].

The MSA results demonstrate that the development of the Twist1 glycine-rich regions is first seen within the fish class (Fig 2). Among this group, only the cavefish (*Astyanax mexicanus*) has acquired glycine residues within glycine-region 2 (right red box). Up to this point in evolution, given the limited number of sequences available, it appears that no glycine residues were present in glycine-region 1. This implies that glycine-region 2 evolved first. The acquisition of glycine residues within glycine-region 1 did not occur up until the evolution of reptiles and birds (left red box), which continued to further develop glycine-region 2. Complete development of both glycine regions does not occur until the evolution of mammals. Interestingly, it is important to note that like Twist2 proteins, amphibian Twist1 sequences do not contain either glycine-rich region, which supports our previous hypothesis that the ancestral Twist sequence is more “Twist2-like”. We excluded the possibility that these *Xenopus* Twist1 sequences could have been Twist2 proteins that were mislabeled when incorporated into the databases because not only did our phylogenetic and metric multidimensional scaling (MMDS) results grouped these sequences within the Twist1 proteins, but also it contains an asparagine (N) residue in the amino-terminus that is characteristic of Twist1 sequences (discussed further below). In addition, studies conducted previously [28] confirm that the *Twist2* gene was lost in the *Xenopus* species but the *Twist1* paralog was retained. Furthermore, based on the alignment, it also appears that Twist2 proteins never developed nor lost the glycine rich regions throughout evolution.

### A new conserved sequence motif with unknown function within the N-terminus of Twist vertebrate proteins

In addition, the MSA demonstrates a highly conserved sequence motif that has not been previously described and is found in the majority of Twist1 and Twist2 vertebrate proteins, particularly amongst the mammalian class where they appear strictly conserved. The motif sequence for Twist1 in mammals is SSSPVSPADDSLSNSEEE while for mammalian Twist2 sequences the motif is SSSPVSPVDSLGTSEEE (depicted by black rectangles in the alignment) (Fig 2). It should be noted that the underlined asparagine residue in the Twist1 motif and the underlined threonine residue in the Twist2 motif are amino acids of great importance since they define the key difference between detecting Twist1 or Twist2 in PROSITE or PHI-BLAST searches (discussed further below). Furthermore, in 2009, Barnes and Firulli compared amino acid sequences of members of the Twist family and these sequence motifs are not found in any other member of the Twist family [2]. The fact that these sequence motifs have persisted throughout evolution, suggests that they must play an important, yet unknown function, that’s specific for the Twist subfamily proteins. Based on the MSA the motifs (in PROSITE format) are as follows:

Twist1: [GSA][SNR]SP[VEA]SP[AV]D**D**S[LVA][SG]NSE[EG]E
Twist2: S[SG][SC]P[VG]SPVDS[LV][VG]TSEEE

The variability seen in the motifs comes from the fish, amphibian, reptile and avian classes. The motifs appear strictly conserved in mammals.

#### SSSPVSP sequence sub-motif

Within the Twist2 proteins studied, this sub-motif is 100% conserved in all vertebrate groups (highlighted in yellow) (Fig 2), with the exception of two species within the fish group (*Gasterosteus aculeatus*, stickleback; and *Oryzias latipes*, medaka). On the other hand, within the Twist1 proteins studied, this sub-motif is 100% conserved only in the mammalian and amphibian groups. In fish, the Twist1 sub-motif shows a signature of [GSA]**SSP**[VE]**SP**, whereas in birds and reptiles it has a signature of **S**[NR]**SP**[VA]**SP**. It should be noted that all Twist2 proteins contain valine (V) and aspartate (D) residues right after the SSSPVSP sequence sub-motif (SSSPVSP**VD**) while most Twist1 proteins contain alanine (A) and aspartate (D) instead (SSSPVSP**AD**). Interestingly, like Twist2 proteins, amphibian and fish Twist1 proteins (with the exception of stickleback) have a valine residue after this sub-motif, which further supports our hypothesis that the more primitive sequences were more “Twist2-like”. Therefore, we can summarize the signature of this sequence sub-motif for all vertebrate Twist1 and Twist2 species studied here as: [GSA][SGNR][SC]**P**[VGEA]**SP**[VA]**D**. A PROSITE scan [31] against the SwissProt database revealed only 16 hits in proteins classified as either Twist1 or Twist2.

#### SEEE sequence sub-motif

This second sequence sub-motif is rich in acidic residues and is 100% conserved in all Twist1 and Twist2 vertebrate proteins except in Twist1 fish, where the majority of the species have the second glutamate (E) substituted for a glycine (G) residue (highlighted in yellow) (Fig 2). The motif signature can be summarized as **SE**[EG]**E**. It remains to be determined whether this acidic region has any functional role. It is important to note that all Twist2 proteins contain a threonine (T) residue (position 17 in mammalian sequences) before the SEEE conserved sequence sub-motif (TSEEE) while all Twist1 proteins have an asparagine residue (position 18 in mammalian sequences) instead (NSEEE), depicted by a red arrow on top of the alignment (Fig 2) [32]. We can describe this second sub-motif as [TN]**SE**[EG]**E** for all vertebrate Twist1 and Twist2 species studied here. A PROSITE scan of the motif [TN]-S-E-[EG]-E against the Swiss-Prot database shows hits in 1,124 proteins, including some Twist1 and Twist2 proteins. The threonine and asparagine residues before this particular sub-motif are of great importance because when searching for Twist1 or Twist2 sequences in public databases they can be used to properly differentiate Twist1 sequences from Twist2 sequences in cases where these have been annotated incorrectly, particularly, when searching for primitive Twist1 sequences (i.e. fish and amphibians), which lack the glycine-rich regions and are of approximate same length as Twist2 sequences (160a.a.). These can easily be mistaken for Twist2 sequences; however, the presence of the asparagine residue allows for the proper classification of such a protein sequence.

#### The signature motif

When scanning PROSITE against the Swiss-Prot and Trembl databases using the two sub-motifs, we find 120 proteins categorized as Twist1, Twist2, Twist-related protein1, Twist-related protein2 or uncharacterized proteins. The latter all show sequence length and motif ordering that indicates they probably are Twist1 or Twist2. We therefore hypothesize that we may have found a Twist protein motif signature in the amino terminus region. The typical locations are amino acids 5–11 of the sequence for the [GSA][SGNR][SC]**P**[VGEA]**SP**[VA]**D** sub-motif, and amino acids 17–21 or 18–22 for the [TN]SE[EG]E sub-motif for Twist2 or Twist1 respectively. The typical length for the sequences are approximately 160 amino acids for the Twist2 or Twist2 related and approximately 200 amino acids for the Twist1 or Twist1-related. All uncharacterized proteins were within these ranges.

From the MSA of Twist1 and Twist2 we can construct a motif in PROSITE format as follows:

[GSA][SGNR][SC]**P**[GVEA]**SP**[VA]**D*D*S**[LVA][VGS][TN]**SE**[EG]**E**

This motif only retrieves 21 Twist1 type proteins. If we remove one of the aspartates for a motif as follows:

[GSA][SGNR][SC]**P**[GVEA]**SP**[VA]**DS**[LVA][VGS][TN]**SE**[EG]**E**

we only retrieve 100 Twist2, Twist-related protein2, or uncharacterized proteins with Twist2 features. By removing residues from either extreme of the motif sequence, sequentially and one at a time, we obtained the same results for separating the Twist1 and Twist2 proteins as above as follows:

Twist1: **P**[VEA]**SP**[VA]**DDS**[LVA][SG]**NSE**
Twist2: **P**[GV]**SPVDS**[LV][VG]**TSE**

These last two motifs represent the **signature motif** for the Twist1 and Twist2 proteins. It should be noted that the second aspartate residue in the Twist1 motif (missing in the Twist2 motif) could also be a defining difference between detecting Twist1 or Twist2 in PROSITE or PHI-BLAST searches. However, this only applies to higher-class vertebrate Twist sequences (i.e. from reptiles to mammals) since the more primitive Twist1 sequences do not contain a second aspartate residue (Fig 2). The development of the second aspartate residue in Twist1 sequences does not occur up until the evolution of reptiles, around the same time that acquisition of glycine residues within glycine-region 1 first occurred. But the presence of either an asparagine or threonine residue in the motif is the defining key difference between detecting the two paralogs. Similarity searches using the PHI-BLAST algorithm from the BLASTP suite show that using the Twist1 signature motif with the human Twist1 protein sequence retrieves 60 sequences of fragments of Twist1 type proteins and no Twist2 type; conversely the human Twist2 protein sequence retrieves only Twist2 proteins and some Twist1 sequences, which we believe may be incorrectly annotated, because even though the total protein length is 160 amino acids, rather than close to 200, they have the 100% conserved threonine at position 17 (data not shown).

In addition, we used Lichtarge's evolutionary trace method, which is a more sophisticated model used for the identification of regions in proteins that diverge after gene duplication [33,34]. This method helps to identify important amino acid residues that are absolutely conserved in both proteins (depicted by boxes) or that are determinants or class-specific for a particular paralog protein (depicted by an X) (S2 Fig). Briefly, as described in [34] the method generates a ‘trace’ when it compares the consensus sequences for groups of proteins that originate from a common node in a phylogenetic tree and it characterizes them by given them a common evolutionary time cut-off (ETC). It also classifies each residue as either: ‘class-specific’ for residues that occupy a strictly conserved location in the sequence alignment, but that differ in the nature of their conservation between both paralogs. “The information obtained by the evolutionary trace (ET) method can then be mapped on to known protein structures” [34]; however, since there is no Twist structure, the identification of important amino acids aid in determining class-specific residues. Based on the ET results, the residues that are depicted as X are class-specific residues, and coincide with the residues previously described using the PROSITE server as key differentiating residues such as the Asparagine (for Twist1) and Threonine (for Twist2). The trace discovered the same motif in the disorder region that we are proposing ad that hasn’t been described before (S2 Fig.). This demonstrates that the most important signature motifs found through PROSITE are consistent with the results obtained with the ET method as the same results are seen by both approaches, which further supports our evolutionary claims.

### Phosphorylation sites within the SSSPVSP and SEEE sequence sub-motifs

Next, we determined whether any of the serine residues found within the SSSPVSP and SEEE sequence sub-motifs were possible targets for post-translational modifications such as phosphorylation. We looked at consensus sequences for serine/threonine phosphorylation and found several protein kinases whose consensus site specificity coincides with serine residues found in our sequences (Table 3) [35]. Only two protein kinase GSK3 (Glycogen synthase kinase-3) and BARK (beta-adrenergic receptor kinase) have phosphorylation sites within the SSSPVSP motif. However, only BARK is specific for Twist2 since the amino acid sequence surrounding the phosphorylation site in Twist1 differs from those found in Twist2 (Table 3). On the other hand, even though kinases LKB1 (also known as STK11 (serine threonine kinase 11)) and CK2 (Casein kinase-2) do not have phosphorylation sites within the SEEE motif per se, they do target the threonine (T) residue before this sequence. As mentioned above, this threonine residue is only present in Twist2 proteins (Twist1 proteins contain an asparagine (N) residue instead), hence making these kinases specific for Twist2. In general, these findings suggest that serine/threonine residues within these two conserved sub-motif sequences may play an important role in Twist protein function that requires their phosphorylation.

**Table 3:** Phosphorylation sites within SSSPVPS and SEEE sub-motifs.

Enzyme	P-site Consensus Sequence	P-Site Sequence	P-Site	Human Protein
GSK3	pS-X-X-X-pS-P	MMQDVS**S**SPVSPADDSLSNSEEE	S7	Twist1
GSK3	pS-X-X-X-pS-P	MEEGS**S**SPVSPVDSLGTSEEE	S6	Twist2
BARK	X-E-X-pS-X-X	MEEG**S**SSPVSPVDSLGTSEEE	S5	Twist2
LKB1	X-L-X-pT-X-X	MEEGSSSPVSPVDSLG**T**SEEE	T17	Twist2
CK2	pS/T-X-D/E-D/E/pS-D/E	MEEGSSSPVSPVDSLG**T**SEEE	T17	Twist2

Consensus phosphorylation site specificity of protein-Ser/Thr kinases was obtained from Kinexus Bioinformatics. Bold amino acids = Target Phosphorylation (P-) site; Underlined amino acids = important amino acids surrounding the target S/T needed by the enzyme for recognition; pS/T = phosphorylated Ser/Thr; X = any residue.

### The N-terminal and C-terminal regions of TWIST proteins are predicted to be disordered

**Fig 3:**
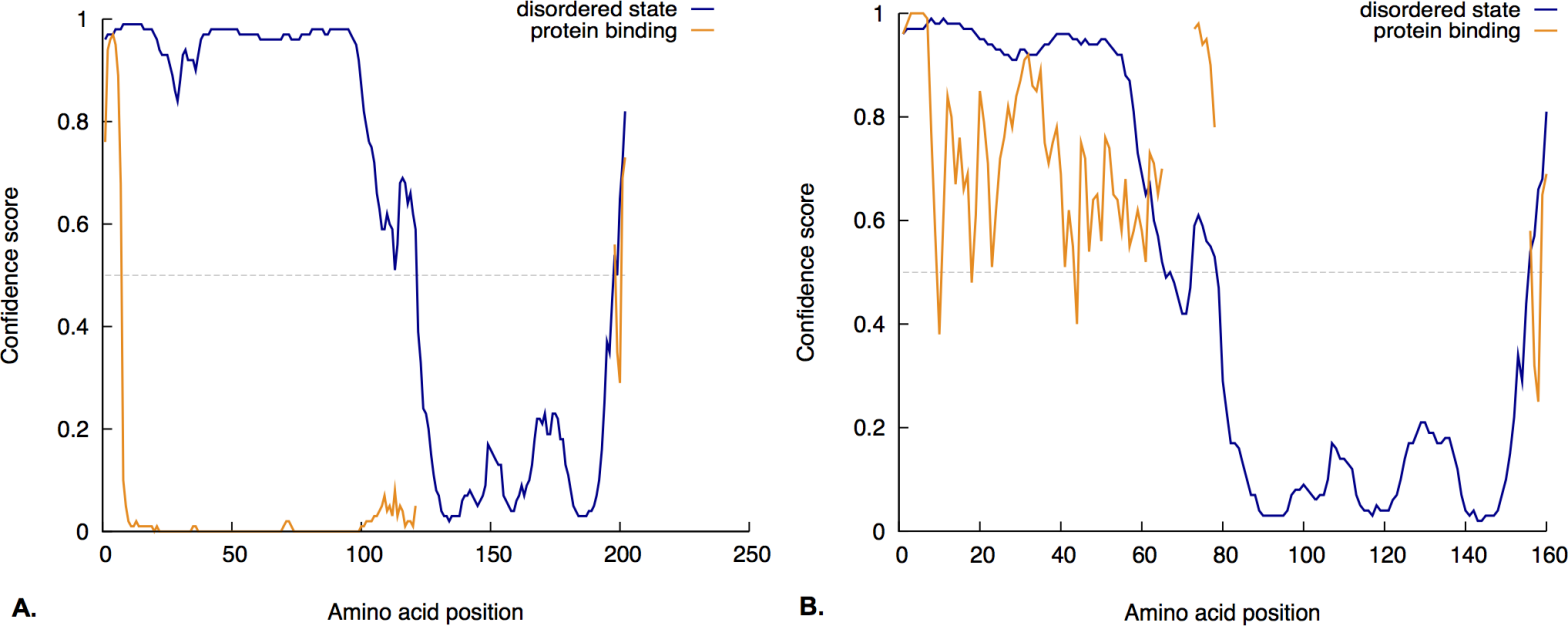
Intrinsic disordered domain and function analysis of TWIST protein sequences. **A) Disordered domain analysis of TWIST1.** Both, the N-terminus and C-terminus of Twist1 are predicted to be in a disordered region as depicted by the blue line. More importantly, it predicts that approximately the first and last 12 amino acid residues of the N-terminus and C-terminus, respectively, have the potential to bind proteins (orange line). **B) Disordered domain analysis of TWIST2.** The entire N-terminus and C-terminus are predicted to be in a disordered state (blue line) and associated with protein binding (orange line). X-axis depicts the amino acid position and y-axis the confidence score. The FFpred web server was used to predict disordered domains and function.

**Fig 4:**
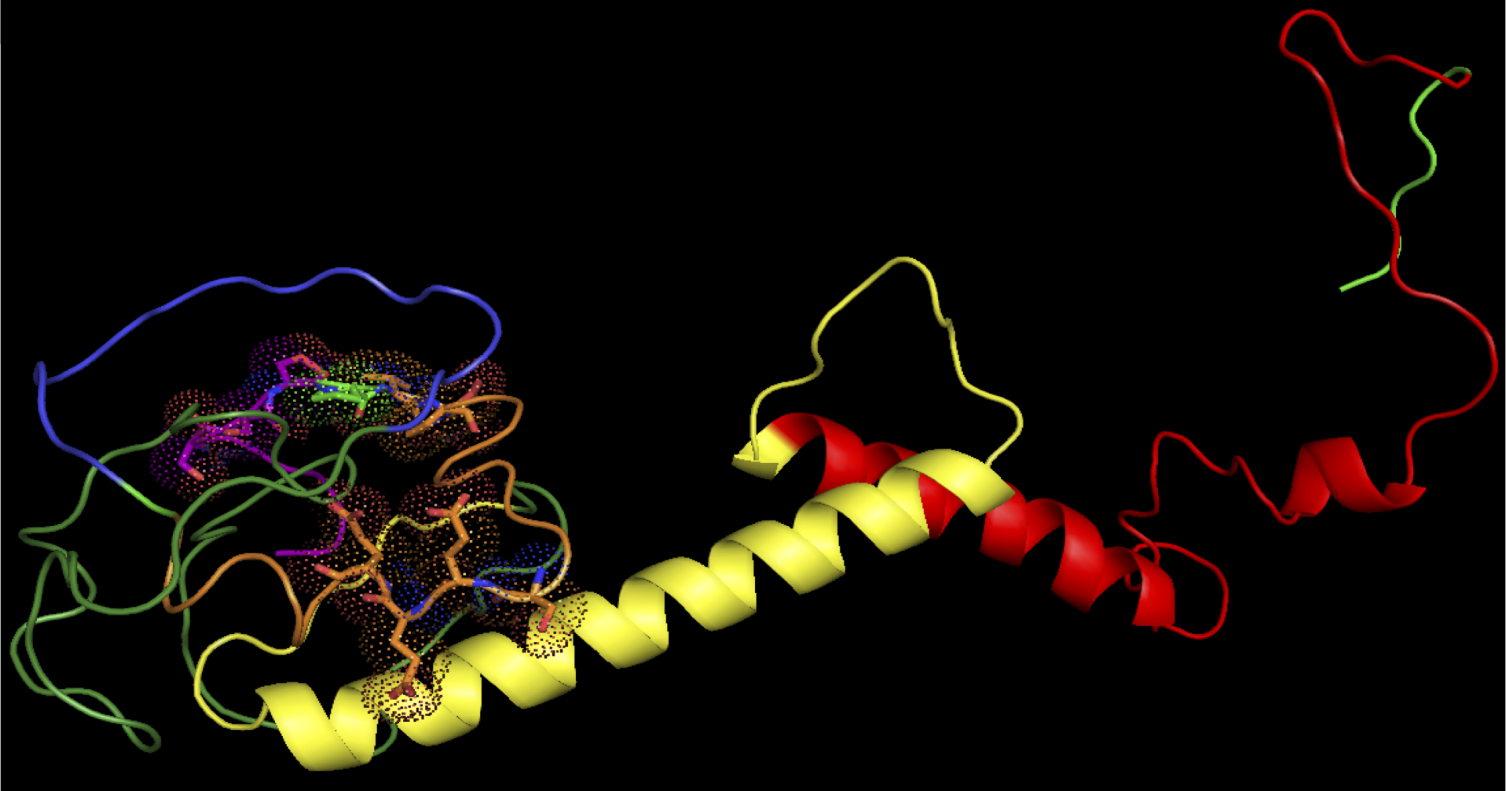
Three-dimensional view of structure prediction of TWIST protein. The Helix-Loop-Helix is represented by the colors yellow and red. The N- and C-terminal regions contain disordered domains. The Phyre2 server was used for structure prediction. Pymol was used for visualization of the predicted structure.

**Fig 5:**
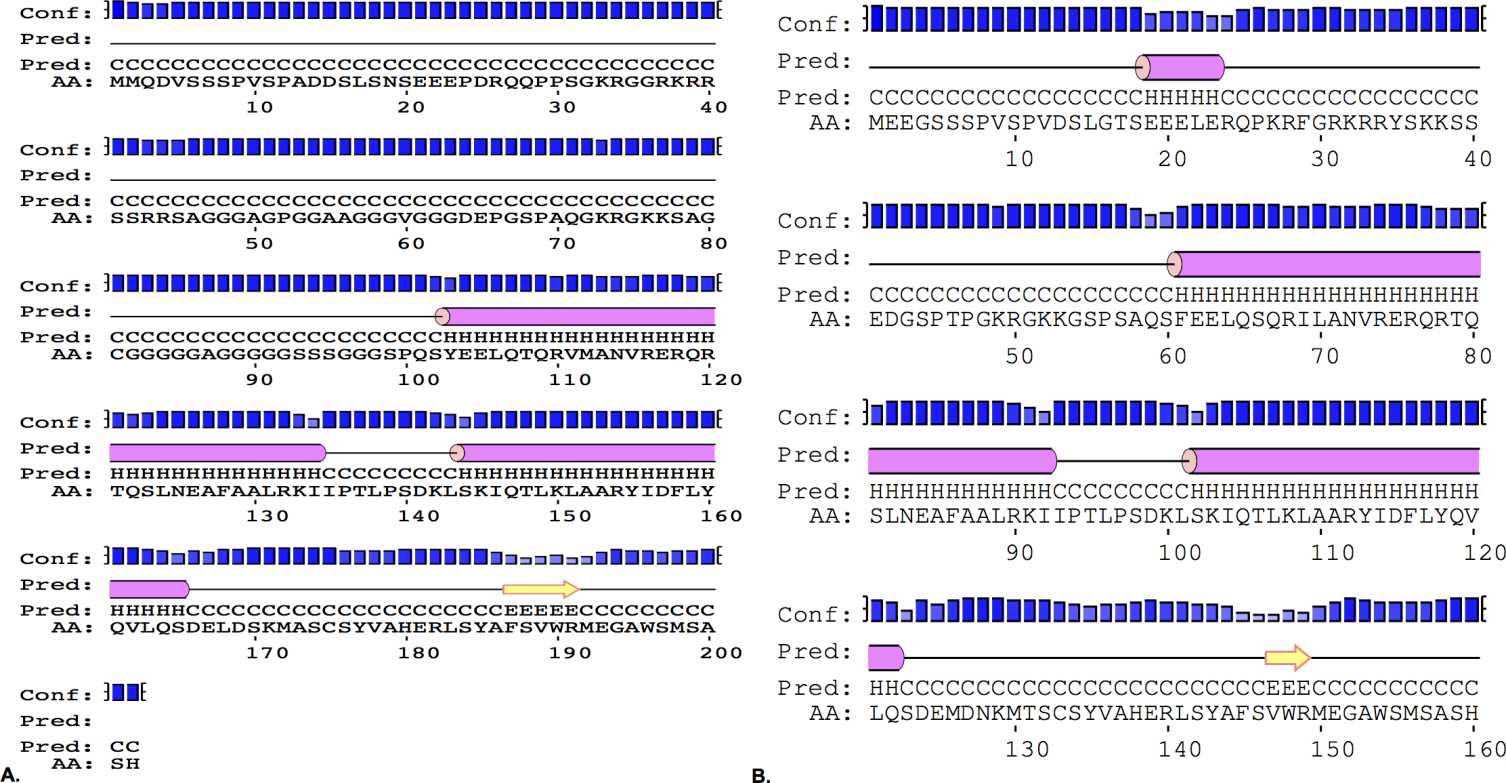
Secondary structure prediction of TWIST proteins. **A) Structural characteristics of TWIST1 protein. B) Structural characteristics of TWIST2 proteins.** The analyses show that both TWIST1 and TWIST2 sequences have a disordered amino terminus domain as expected. It also predicts a high degree of structure for the bHLH domain, and the carboxy terminus in a disordered/flexible region. Pink tube = helix; Yellow arrow = strand; Black line = coil; Blue bars = confidence of prediction; Pred = predicted secondary structure; A.A. = target sequence. The FFpred web server was used for structure prediction.

Usually, the functional domains of a protein are highly structured, and this is seen in their three-dimensional structure. However, it has also been documented that protein domains with functional importance can be found in disordered regions rather than the typical tightly folded domain [36,37]. As described in [36], a disordered domain usually means that it lacks regular secondary structure yet contains a great amount of flexibility in the polypeptide chain. These disordered regions can contain functional sites, and although most are unstructured when in solution, they can become ordered once they come in contact with another molecule [38,39]. Globplot studies conducted by Maia et al. [40] demonstrated that amino acid residues 3–102 (N-terminus) and 193–200 (C-terminus) of TWIST1 are found in a disordered region. Taking this information into consideration, we wanted to explore whether these disordered regions were associated with a particular molecular activity using the function prediction (FFpred) web server [41]. FFpred results also predict that the N-terminus and C-terminus of TWIST1 protein are found in a disordered state (blue line, Fig 3A). More importantly, it predicts that approximately the first and last 12 amino acid residues of the N-terminus and C-terminus, respectively, have the potential to bind proteins (orange line, Fig 3A). Interestingly, the SSSPVSP sub-motif is found within the first 12 amino acid residues of the N-terminus. Like TWIST1, TWIST2’s N-terminus and C-terminus are also predicted to be in a disordered state (blue line, Fig 3B). However, its entire N-terminus (with the first 12 amino acids having the highest confidence score) and approximately the last 12 amino acids of its C-terminus are predicted to be associated with protein binding (orange line, Fig 3B). Therefore, this suggests that the SSSPVSP sub-motif has the potential to bind proteins. Furthermore, since there is no Twist structure available, the Phyre2 Structure Prediction server was used to obtain a model of the structure of TWIST using comparative homology modeling [42]. The Phyre2 results predict that the N- and C-terminal regions are disordered (Fig 4). In addition, FFpred also gave a different visual format of the secondary structural characteristics of the human TWIST1 and TWIST2 sequences, which are similar to the secondary structure prediction made by Phyre2 (Fig 5).

**Fig 6:**
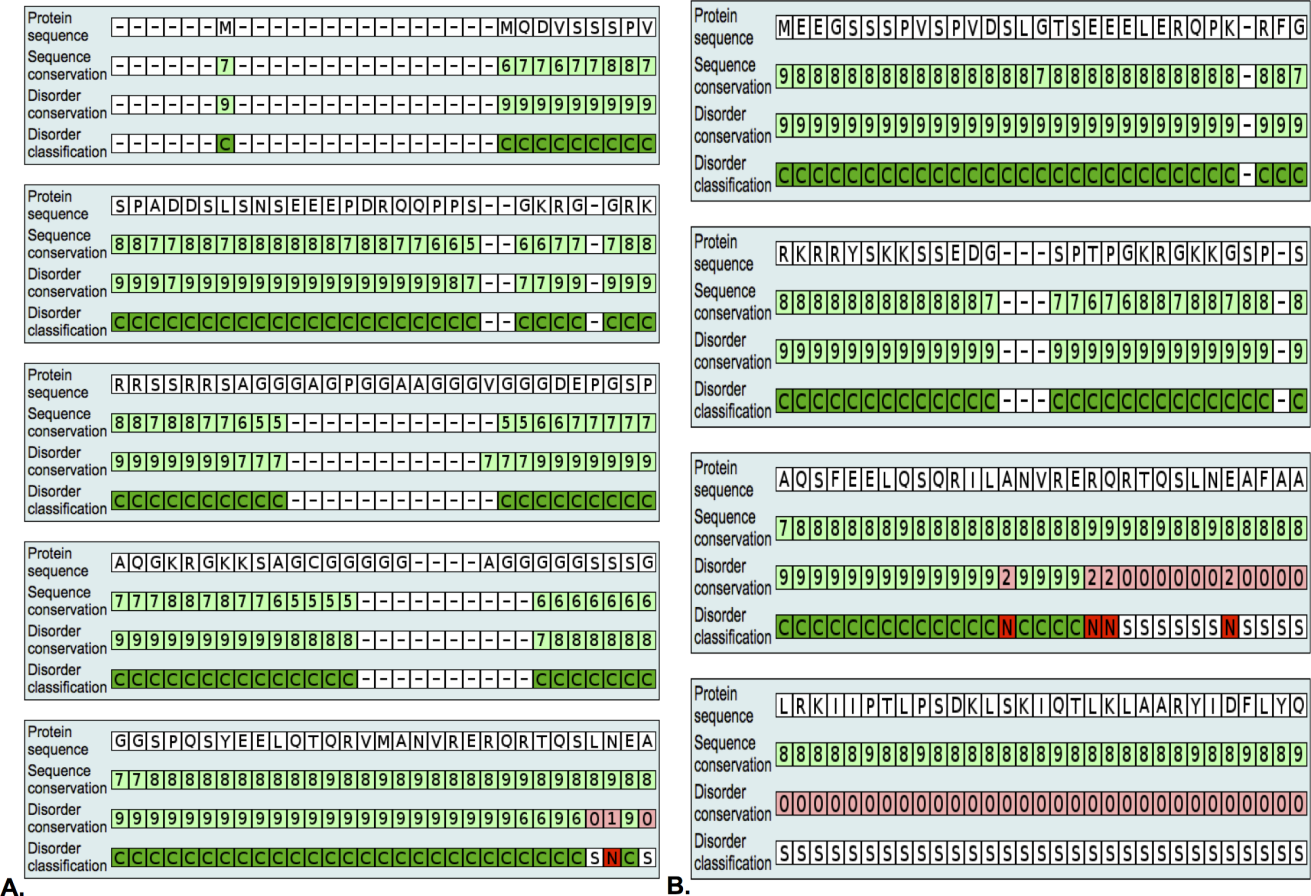
Evolutionary Conservation of Intrinsic Protein Disorder. **A) Disorder conservation prediction of Twist1 proteins.** The disorder feature found in the N-terminus region of Twist1 is evolutionary conserved. **B) Disorder conservation prediction of Twist2 proteins.** The disorder feature found in the N-terminus region of Twist2 is evolutionary conserved. Disorder classification: Structured (white, S) if there is zero disorder. Constrained (green, C) if both the sequence and disorder conservation scores are 5 or greater. Non-conserved (red, N) if the disorder conservation score is below 5. (-) means gap. DisCons tool was used to asses for evolutionary conservation of intrinsic protein disorder.

Furthermore, as stated by Mihaly Varadi (2015), when the amino acid sequence of an intrinsic disordered protein or disorder region (IDP and/or IDR) is analyzed from an evolutionary context, it can yield new information on the functional role of disordered regions and sequence elements, particularly when one combines the information obtained from analyzing the conservation of sequence and disorder [43]. Taking this information into account, we next determined whether the disorder feature found in the N-terminus region was evolutionary conserved using the DisCons tool. As demonstrated in Fig 6, the disorder feature found in the N-terminus as well as the amino acid sequence are found to be evolutionary constrained and not highly divergent. The fact that both, disorder and amino acid sequence have been conserved across Twist homologs throughout evolution highlights this structurally disordered segment as being potentially functional; hence of great value for the function of the proteins, which further supports the importance of studying this region.

### Detailed examination of the N-terminus of TWIST1

**Fig 7:**
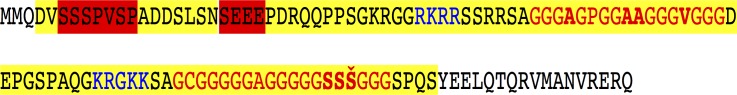
Amino terminus region of human TWIST1 protein. The disordered region is highlighted in yellow and comprises of amino acids 3–102. The first glycine-rich motif contains aliphatic amino acids when compare to the second glycine motif, which contains serine (S) polar residues (colored in red). Highlighted in red are the new conserved sequences found. Colored in blue are the two Nuclear Localization Signals (NLS). Underlined amino acids (108–119) depict the basic domain of the protein. Š = serine residue predicted as targeted for phosphorylation by GSK3 kinase.

Rogelj et al. [44] examined the glycine-rich regions of individual RNA Binding Proteins (RBPs) and found that within these regions there were adjacent polar residues. They suggested that the interaction of glycine-rich regions with RNA or other proteins, or the likelihood of their post-translational modification, is possibly due to the presence of these additional residues within these regions [44]. Taking this into account, we decided to conduct a more detailed examination of the amino terminus region of TWIST1. As seen in Fig 7, the glycine-rich regions are found in a disordered region (highlighted in yellow). When looking at the first glycine-rich motif, we found there are mostly nonpolar residues, alanine (A), valine (V) and proline (P). Therefore, this first glycine-rich region is a G-A region that is rich in amino acids with aliphatic side chains. It is worth noting that the Epstein-Barr nuclear antigen-1 (EBNA-1) has a repeat that is rich in glycine-alanine, demonstrated to be antigenic [45]. However, further examination of proteins containing G-A regions is required to determine their possible role. Also, since this region is rich in aliphatic residues, it would be interesting to examine what changes in conformation occur when bound to DNA and/or the partner in the dimer to see if one can detect conformational changes indicative of structural ordering.

When examining the residues found within the second glycine-rich region, a stretch of polar serine (S) residues is present. Therefore, this region is a G-S region. Interestingly, a similar G-S region is also found in the Sx1 RNA binding protein [44], which raises the question of whether this region is used in RNA binding. In addition, it is not known whether these serine residues are determining the post-translational state of this particular region. However, the third serine residue (Ŝ) is part of a GSK3 kinase consensus phosphorylation specificity site (pS-X-X-X-pS-P) (Table 3, Fig 7) [35]. This suggests that at least this particular serine might be important in post-translational modifications. It is likely that both the G-A and G-S regions have different functions, since each region might be interacting with different proteins, RNA, or even be regulated differently by phosphorylation, as has been suggested [44].

### Phylogenetic and metric Multi-Dimensional Scaling analysis

**Fig 8:**
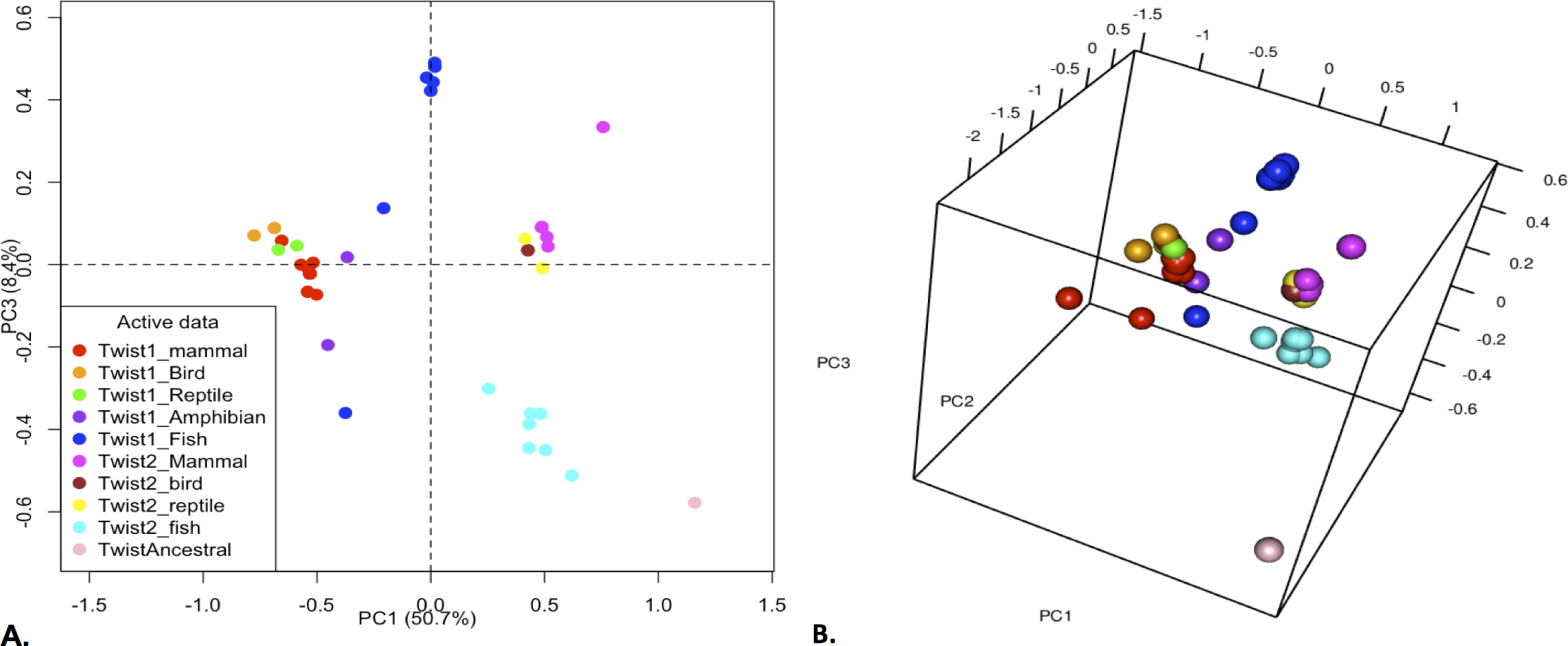
Phylogenetic analysis of vertebrate Twist proteins. The outgroup Twist protein (Twist_BB) is located at the root of the tree. The analysis shows that the Twist vertebrate family underwent a gene duplication event that split Twist proteins into two main clades: Twist1 and Twist2. Different vertebrate species are represented by the following colors: Magenta (Twist2 mammals), Yellow (Twist2 reptiles), Brown (Twist2 birds), Cyan blue (Twist2 fish), Blue (Twist1 fish), Purple (Twist1 amphibians), Green (Twist1 reptiles), Orange (Twist1 birds), Red (Twist1 mammals). Numbers on each node display bootstrap values. The TrimAl package in the gappyout mode was used to trim the alignment. A maximum likelihood tree was constructed using PHYLIP (MPIproml) with 1000 bootstrap replicates. The program FigTree was used to visualize the phylogenetic tree. Sequence names used represent the common name of the species to which they belong.

In our maximum-likelihood phylogenetic tree, the principal clades are supported with bootstrap values greater than 45 or 50% (Fig 8). These bootstrap values have been considered acceptable for many proteins such as ABC transporters [46]. Nonetheless, the split for each vertebrate class, particularly the mammalian class, were well supported, with high confidence, as shown by the high bootstrap values. However, at the level of the leafs, the bootstrap values are low, probably due to the fact that there is very high sequence similarity, which causes the phylogenetic signal to be low. In some cases, but particularly within the mammalian Twist1 and Twist2 groups, their sequences are too similar to obtain good support for the nodes that are close to the leafs. Following Castanon and Baylies [1], we used the Twist_BB sequence as the outgroup for this analysis, as it is deemed closer to the ancestral sequence. The phylogenetic analysis places the Twist_BB sequence between the Twist2 fish and reptile groups and not between fish Twist1 and Twist2, as one would expect. Twist2 sequences, particularly those in the fish group, are placed closest to the Twist_BB sequence. This may be caused by some long-branch attraction problem, or due to the sparse sequence record available to carry out the analysis. Furthermore, the tree also clearly shows that the appearance of the Twist1 sequences came from the Twist2 fish group, when we can speculate that a gene duplication occurred.

Metric multidimensional scaling (MDS) constructs a matrix of distances between aligned homologous proteins, a process used by distance-based phylogenetic methods [47]. The distance matrix used by distance-based phylogenetic methods to acquire information on the evolution of protein families maybe used in metric multimensional scaling (MDS) that when applied to protein families, it can be used to obtain information that is complementary from the information obtained from tree-based methods; hence allowing for a better visualization of the evolutionary drift of protein sub-families and the comparison of orthologous sequences in sequence space. [47–49]

**Fig 9:**
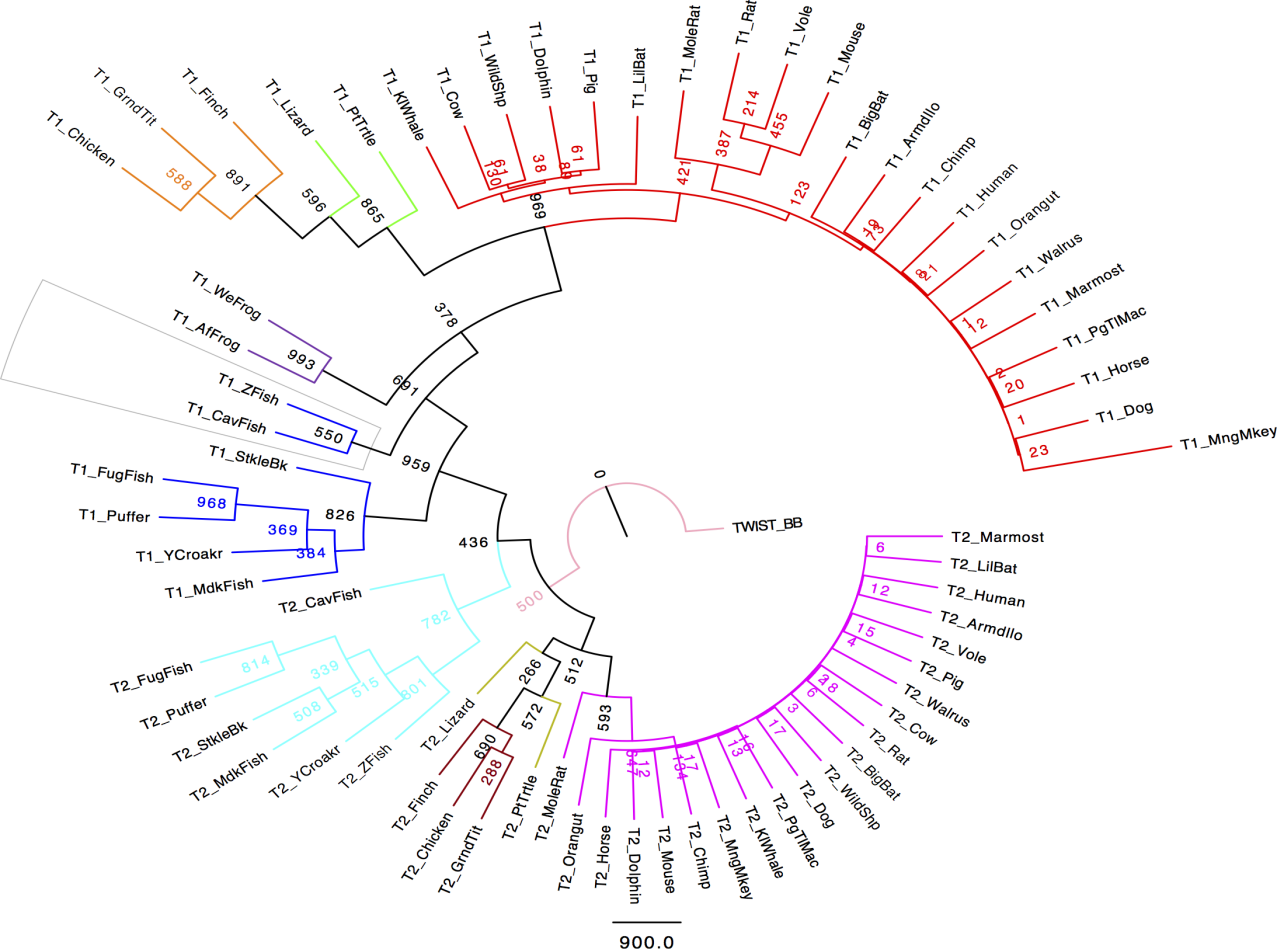
Largest Principal Components of the MMDS Analysis for the Twist1 and Twist2 proteins. A) Two-dimensional plot of principal components 1 and 3 of the MMDS analysis. B) Three-dimensional plot of the most important principal components determined from the MMDS analysis. It can be seen that the Twist2 are closer to the Twist_BB in sequence space. Also, that the Twist1 sequences show more evolutionary drift over sequence space. Different vertebrate species are represented by the following colors: Magenta (Twist2 mammals), Yellow (Twist2 reptiles), Brown (Twist2 birds), Cyan blue (Twist2 fish), Blue (Twist1 fish), Purple (Twist1 amphibians), Green (Twist1 reptiles), Orange (Twist1 birds), Red (Twist1 mammals).

When one looks at the analysis of sequence space with metric multidimensional scaling **(**MDS) (Fig 9), the Twist_BB protein, which is closer to the ancestor, is located in a region of the sequence space separated from the other sequences. The Twist2 proteins are more tightly clustered together in sequence space, and are closer to Twist_BB in sequence space (Fig 9A). Fish Twist2 proteins are separated from the other vertebrate groups, and show more divergence, as expected given their higher variability (Fig 9B). The mammalian, avian and reptile Twist2 sequences are grouped in a very tight cluster. Vertebrate Twist1 sequences are grouped separately from Twist2 sequences, and they showed a bigger degree of evolutionary drift in sequence space (Fig 9A). Within Twist1 sequences, fish Twist1 and to a lesser extent amphibian Twist1 showed a larger degree of divergence than other Twist1 species. Mammalian, avian and reptile species are clustered closer to each other, as they show a higher degree of conservation even within the disordered regions. From these results we can see that the Twist1 proteins are evolving and changing faster than the Twist2. Furthermore, we infer that Twist proteins in fish are evolving faster than those of the other vertebrate classes.

## Discussion

Twist proteins are multifunctional transcriptional factors in terms of their interactions with other macromolecules. However, as in the case of RunX2 [19], ADD1 [20], MEF2 [21,23], p300 and pCAF [22], PGC-1α [26], and p53 [25], there are certain interactions where data demonstrates that there are other elements or domains, besides the common bHLH domain, involved in the protein-protein interactions with Twist transcription factors. When looking at the amino terminus region of Twist vertebrate proteins, new conserved sequence motifs were found in the amino terminus region of all Twist1 (SSSPVSPADDSLSNSEEE) and Twist2 (SSSPVSPVDSLGTSEEE) mammalian species and in the majority of the other vertebrate groups. The function of these conserved sequences, though they are very short acidic domains, remain yet to be described. However they must play an important function since they have been maintained throughout evolution, and consensus site specificity for serine/threonine phosphorylation of some kinases coincide with serine/threonine residues within the first sub-motif SSSPVSP and near the second sub-motif SEEE. It is worth noting that a study conducted by Jiateng Zhong (2013), suggests that IKKβ (not identified by our searches) could phosphorylate Twist1 at multiple sites within the first 30 amino acids of Twist1’s N-terminus to trigger Twist destruction by β-TRCP in HeLa cervical cancer cells [50]. More specifically, mutations of Ser7, Ser8, Ser11, Ser16 and Ser20 to Ala (all but Ser16 found within the first and second sub-motifs) decreased phosphorylation by IKKβ. However, the contribution of each individual phosphorylation site in the destruction of Twist by β-TRCP has yet to be determined. Interestingly though, all of these serine residues are also conserved in Twist2 protein, which suggests that Twist2 could also be a target of IKKβ kinase. However, more *in-vitro* kinase assays are needed in the N-terminus of Twist proteins, particularly that of Twist2, since most studies have focused in Twist1.

Furthermore, we identified a new signature motif for Twist1 proteins: **P**[VEA]**SP**[VA]**DDS**[LVA][SG]**NSE** and for Twist2 proteins: **P**[GV]**SPVDS**[LV][VG]**TSE.** However, it is the presence of either an asparagine (for Twist1) or threonine residue (for Twist2) in the motif, which represents the key difference in the differentiation between the two paralogs. By providing a new Twist protein motif signature in the amino terminus region, and highlighting key amino acid residues within this motif that are characteristic for each paralog protein, we offer a way to reduce Twist1 and Twist2 sequence annotation errors in public databases since they can be used to properly differentiate Twist1 from Twist2 sequences.

Comparison of human Twist1 and Twist2 proteins with a Twist protein that is more like the ancestral protein showed that Twist2 is more similar to the ancestral Twist than Twist1. Multiple sequence alignments of the vertebrate Twist proteins demonstrated that the glycine-rich regions observed in Twist1 sequences were not present early in the evolution of vertebrates. At the beginning of vertebrate evolution, similarly to Twist2 sequences, Twist1 sequences did not contain the glycine-rich regions now present in human Twist1 sequences. Instead, what differentiates Twist1 from Twist2 early in evolution is the presence of an asparagine (N) or threonine (T) residue, respectively, before the serine residue of the SEEE sub-motif sequence. Twist1 glycine-rich regions were acquired later in evolution, perhaps through simple tandem repeat mutations and recombination/duplication events [28]. Acquisition of both regions did not occur at the same time. The second glycine-rich region developed first among the fish vertebrate group, while the first glycine region developed afterwards within the reptiles. Overall, mammalian, avian and reptile Twist1 sequences seemed to demonstrate a higher sequence divergence when compared to Twist2 sequences of the same species. Since Twist2 sequences do not contain the two glycine-rich regions, perhaps these regions allow Twist1 proteins to interact with particular proteins that are not bound by Twist2 proteins [15,28].

The conserved sequence found in both Twist proteins, as well as both glycine-rich regions of Twist1 are found in the disordered domain region of the amino terminus and are predicted to be associated with protein binding. More importantly, the amino acid sequence and the regions predicted to be disordered are shown to be conserved, and not highly divergent. The fact that this region is evolutionary constricted highlights the importance of characterization and function inference of disordered protein regions. Although this still remains a difficult task, computational methods such as the ones presented here that focus at the sequence level, have proven to be relevant for the study of intrinsically disordered proteins and protein regions (IDPs/IDRs) [36,51]. It is of great importance to study and understand the potential functionality of conformational flexibility/disorder since, as described in [52], disordered regions can also provide several advantages to the function of a protein by providing more flexibility in terms of their conformation, a greater surface for protein interactions, exposure of structural motifs used for interaction, and diverse regulation of their function due to post-translational modifications. Therefore, by studying the local amino acid context within the disordered region it is possible to infer the amino acid interactions that can take place and/or to determine the state of the protein chain for example, whether it is found in an extended or collapsed state [43]. Deletion mutagenesis of the N-terminus of TWIST1 and TWIST2 can be another option to better understand the importance of this disordered yet highly conserved region in the function of the protein.

Further experimental studies of glycine-rich domains, particularly G-A and G-S rich regions, are needed to provide insights into their role in protein-protein interactions. The adjacent residues found within these motifs could play important roles not only in post-translational modifications but also in the interaction with other proteins and RNA [44]. Most of the proteins that contain glycine-rich regions are found in plants and RNA binding proteins, and are implicated in a variety of different processes such as transcriptional regulation, signal transduction, development and stress response [53]. Therefore, proteins containing glycine-rich domains in vertebrates could very well be behaving like the proteins found in plants, and/or these domains could be used for RNA binding, which to our knowledge, has not been examined. Understanding the role of these proteins in plants and evaluation of potential RNA binding activity could help us understand their roles in human cell biology, hence, becoming useful biological targets in physiological and biochemical processes.

Our phylogenetic and sequence space analysis helped us to better understand the evolution and degree of evolutionary drift between Twist1 and Twist2 sequences. The phylogenetic analysis presented here was done based on a trimmed MSA version in which the most highly diverged segment of the N-terminus (the glycine-rich regions) were taken out, thus increasing the extent to which the phylogenetic inference and evolution history can be inferred. The different branch lengths observed in the phylogenetic tree suggested that the evolution of Twist1 and Twist2 paralogs has been different throughout time, as has been described [1,47]. Both, the MDS and phylogenetic analyses support our finding that Twist2 sequences are more similar to the ancestor Twist protein while suggesting that the Twist1 proteins in general, and particularly those within the Fish group, are undergoing more evolutionary drift than the Twist2 fish sequences and other vertebrates in general. In addition, the phylogenetic tree also suggests that the evolution of Twist1 sequences came from the fish Twist2 group, but inclusion of more species that have both paralog sequences, particularly from reptiles and amphibians, are needed to obtain a more comprehensive analysis.

## Conclusions

In conclusion, the present study provides important bioinformatic information concerning the amino-terminus region of Twist proteins that highlights how a shift of focus towards this region is imperative in order to fully comprehend its functional significance. It was determined that the apparition of both glycine-rich regions of Twist1 is first seen among the reptiles and a new conserved sequence motif with unknown function yet predicted to be associated with protein binding was found in the intrinsically disorder amino-terminus of both proteins, which cannot be described as being highly divergent because it is evolutionary conserved. Evolution tends to conserve motifs conformational features that are important in the function of the proteins, and tends to evolve new sequences and structure features that will improve its function. Therefore, in order to fully comprehend and differentiate the modes of action of Twist1 and Twist2 further analysis of the conserved sequences and detailed examination of both glycine-rich regions among the Twist1 sequences is of great importance since not only can these sites be used for interaction with other proteins but they can also provide for additional regulation of their function by post-translational modifications. However, more information that directly compares the protein-protein interactions exerted by the N-terminus of Twist1 and Twist2 is needed in order to better understand the functional role of this region. In addition, without a Twist 3-dimensional structure it is hard to determine the type of conformation these regions acquire once in contact with other domains and proteins. Structural analysis of these regions of the protein would provide insights into the type of surface area and interactions they provide once in contact with another protein. It can also provide information as to the exact position and spatial proximity of the functional groups of amino acids in these regions that may facilitate protein-protein interactions. Nonetheless, sequence based investigation of intrinsic disorder such as the evolutionary conservation of sequence and conformational flexibility aid in the prediction of functional sites and/or the functional role of intrinsic disorder by analyzing the characteristics of the enriched amino acids that lie within such regions. Analysis of such distinct sequence characteristics allow in turn for the development of future experimental designs to specifically investigate their functional roles. Finally, when searching in public databases for either Twist1 or Twist2 sequences, the presence or absence of the glycine-rich regions is not enough to discriminate between Twist1 and Twist2 sequences since as mentioned above, early in evolution, Twist1 sequences did not contain the glycine-rich regions. The present study provides a signature motif in the amino terminus region that is particular of Twist sequences that can be used to correctly discriminate between these two paralogs. These findings will aid in improving correct annotations of Twist sequences in public databases and hence reduce miss-identification of Twist paralog proteins.

## Methods

### Taxanomic sampling and sequence dataset

Ortholog and paralog Twist sequences were obtained from the NCBI Protein Database and the UniProtKB database using the BLAST algorithm [30] in the blastp mode with default parameters (Tables 1 and 2). Altogether, 68 Twist protein sequences (35 Twist1 and 33 Twist2 sequences) were identified based on sequence homology in five major vertebrate classes through database searches: 21 Twist1 and Twist2 mammalian sequences: *Homo sapien*s (human), *Pan troglodytes* (chimpanzee), *Pongo abelii* (sumatran orangutan), *Macaca nemestrina* (pig-tailed macaque), *Cercocebus atys* (sooty mangabey monkey), *Callithrix jacchus* (white-tufted-ear marmoset), *Mus musculus* (mouse), *Rattus norvegicus* (rat), *Microtus ochrogaster* (prairie vole rodent), *Heterocephalus glaber* (naked mole-rat), *Canis familiaris* (dog), *Bos taurus* (bovine), *Ovis orientalis musimon* (wild sheep), *Sus scrofa* (pig), *Lipotes vexillifer* (yangtze river dolphin), *Odobenus rosmarus divergens* (walrus), *Orcinus orca* (killer whale), *Myotis lucifugus* (little brown bat), *Eptesicus fuscus* (big brown bat), *Equus przewalskii* (wild horse), *Dasypus novemcinctus* (nine-banded armadillo); 3 Twist1 and Twist2 bird sequences: *Gallus gallus* (chicken), *Taeniopygia guttata* (zebra finch), *Pseudopodoces humilis* (tibetan ground-tit); 7 Twist1 and Twist2 fish sequences: *Astyanax mexicanus* (Mexican tetra blind cavefish), *Danio rerio* (zebrafish), *Oryzias latipes* (medakafish), *Takifugu rubripes* (fugufish), *Larimichthys crocea* (yellow croaker), *Gasterosteus aculeatus* (stickleback), *Tetraodon nigroviridis* (pufferfish); 2 Twist1 and Twist2 reptile sequences: *Anolis carolinensis* (lizard), *Chrysemys picta bellii* (western painted turtle); and 2 Twist1 amphibian sequences: *Xenopus laevis* (African clawed frog), *Xenopus tropicalis* (Western clawed frog). Species were chosen because they represented major vertebrate groups and because they each contained Twist1 and Twist2 paralogs that could be used for comparison. Although a Twist2 paralog is lost in the *Xenopus* species, we decided to include Twist1 *Xenopus* sequences because they provided important information. The nomenclature for the Twist family members was based on orthology to the mammalian *Twist1* and *Twist2* genes and according to the name of the species to which they belonged [28]. In addition, 2 ancestor twist-like protein sequences were found within the amphioxus group: *Branchiostoma belcheri* (Belcher's lancelet) and *Branchiostoma floridae* (Florida lancelet). The sequences used in this analysis were chosen only if their bHLH identity percentage was above 89% as described in [28], and if the overall protein sequence shared >75% of identity when compared to human twist sequences.

### Multiple Sequence Alignments and Motif Analysis

The PSI-COFFEE and the T-COFFEE suite of programs [54,55] with default parameters were used to construct the MSAs. The software program GeneDoc or the T-COFFEE server was used for visualization of the alignments [54,56]. The PROSITE server [31] and the Lichtarge's evolutionary trace method as implemented in the University of Cambridge Department of Biochemistry Server TraceSuite II by [34] were used for the identification of regions in proteins that diverge after gene duplication. We performed phylogenetic analysis on a trimmed MSA version in which the least conserved segments of the N-terminus (the glycine-rich regions) and regions with large groups of gaps were removed, in order to improve the quality and robustness of the phylogenetic trees and of the evolution history that can be inferred [57].

### Disordered Domain Analysis and Secondary Structure Prediction

The programs in the FFpred-server were used to assess for any disordered domains and for function prediction [41]. The Phyre2 Structure Prediction server [42] was used to construct a representation of the secondary structure of the Twist1 protein and Pymol [58] was used for visualization of the predicted structure. The DisCons web server [43] was used for Disorder Conservation analysis using default parameters and a BLOSUM80 similarity matrix.

### Phylogenetic Analysis

The MSA results were used to construct the phylogenetic tree. The MSA was trimmed using the program trimAl in the gappyout mode [59]. The resulting trimmed alignment was used to build a phylogenetic tree using maximum likelihood and 1000 bootstrap replicates. The parallel version of the PHYLIP maximum likelihood method for proteins—MPIproml was used to construct the tree [60]. The phylogenetic tree was viewed using the program FigTree [61].

### MMDS Analysis

The trimmed MSA was used as input for metric multi-dimensional scaling analysis. The MMDS analysis was performed with the R package bios2mds [47], using a JTT similarity matrix to construct the distance matrix required for the analysis.

## Supporting Information

S1 Fig A.A. comparison between ancestor Twist *Branchiostoma belcheri* (BB), *Branchiostoma floridae (BF)* and human Twist protiens. *Branchiostoma belcheri* (BB) and *Branchiostoma floridae (BF)*; both share approximately 89% bHLH identity when compared to human Twist sequences. Both ancestor proteins contain approximately three or four glycine surrounding the second glycine-rich region, which further suggest this region evolved first. Overall, when compared with BF, Twist2 shares 68% amino acid identify while Twist1 shares 56%, which further suggests that the ancestor of both Twist paralogs was a “Twist2-like” protein. Below the protein sequence: (*) = Good conserved residues (dark pink); (:) = average conservative mutations (yellow); (.) = semi-conservative mutations (pink); () = non-conservative mutations (purple and green are indicative of badly conserved residues). Twist_BB = *Branchiostoma belcheri* species. Twist_BF = *Branchiostoma floridae*. The alignment was performed with PSI-COFFEE.
(TIFF)

S2 Fig Evolutionary traces for partitions P01–P10, aligned with the amino acid sequences of Twist paralgos. Conserved residues are surrounded by boxes, while class-specific residues, particularly the as Asparagine (for Twist1) and Threonine (for Twist2) are denoted by an X. The ET results also detected the same sequence motif found using PROSITE.
(TIFF)

## References

[1] Castanon I, Baylies MK. A Twist in fate: Evolutionary comparison of Twist structure and function. Gene. 2002;287(1–2):11–22. 11992718 10.1016/s0378-1119(01)00893-9

[2] Barnes RM, Firulli AB. A twist of insight—The role of Twist-family bHLH factors in development. Int J Dev Biol. 2009;53(7):909–24. 10.1387/ijdb.082747rb 19378251 PMC2737731

[3] Jones S. An overview of the basic helix-loop-helix proteins. Genome Biol. 2004;5(6):226 15186484 10.1186/gb-2004-5-6-226 PMC463060

[4] Hebrok M, Wertz K, Füchtbauer EM. M-twist is an inhibitor of muscle differentiation. Dev Biol. 1994;165(2):537–44. 7958419 10.1006/dbio.1994.1273

[5] Lee MS, Lowe GN, Strong DD, Wergedal JE, Glackin C a. TWIST, a basic helix-loop-helix transcription factor, can regulate the human osteogenic lineage. J Cell Biochem. 1999;75(4):566–77. 10572240 10.1002/(sici)1097-4644(19991215)75:4<566::aid-jcb3>3.0.co;2-0

[6] Lee MS, Lowe G, Flanagan S, Kuchler K, Glackin C a. Human dermo-1 has attributes similar to twist in early bone development. Bone. 2000;27(5):591–602. 11062344 10.1016/s8756-3282(00)00380-x

[7] Li L, Cserjesi P, Olson EN. Dermo-1: a novel twist-related bHLH protein expressed in the developing dermis. Dev Biol. 1995;172(1):280–92. 7589808 10.1006/dbio.1995.0023

[8] Tukel T, Šošić D, Al-Gazali LI, Erazo M, Casasnovas J, Franco HL, et al Homozygous nonsense mutations in TWIST2 cause setleis syndrome. Am J Hum Genet. 2010;87(2):289–96. 10.1016/j.ajhg.2010.07.009 20691403 PMC2917720

[9] Marchegiani S, Davis T, Tessadori F, Van Haaften G, Brancati F, Hoischen A, et al Recurrent Mutations in the Basic Domain of TWIST2 Cause Ablepharon Macrostomia and Barber-Say Syndromes. Am J Hum Genet. 2015;97(1):99–110. 10.1016/j.ajhg.2015.05.017 26119818 PMC4572501

[10] el Ghouzzi V, Le Merrer M, Perrin-Schmitt F, Lajeunie E, Benit P, Renier D, et al Mutations of the TWIST gene in the Saethre-Chotzen syndrome. Nat Genet. 1997;15(1):42–6. 8988167 10.1038/ng0197-42

[11] El Ghouzzi V, Lajeunie E, Le Merrer M, Cormier-Daire V, Renier D, Munnich a, et al Mutations within or upstream of the basic helix-loop-helix domain of the TWIST gene are specific to Saethre-Chotzen syndrome. Eur J Hum Genet. 1999;7(1):27–33. 10094188 10.1038/sj.ejhg.5200240

[12] Šošić D, Richardson J a, Yu K, Ornitz DM, Olson EN. Twist regulates cytokine gene expression through a negative feedback loop that represses NF-kappaB activity. Cell. 2003;112(2):169–80. 12553906 10.1016/s0092-8674(03)00002-3

[13] Cheng GZ, Chan J, Wang Q, Zhang W, Sun CD, Wang LH. Twist transcriptionally up-regulates AKT2 in breast cancer cells leading to increased migration, invasion, and resistance to paclitaxel. Cancer Res. 2007;67(5):1979–87. 17332325 10.1158/0008-5472.CAN-06-1479

[14] Shiota M, Izumi H, Onitsuka T, Miyamoto N, Kashiwagi E, Kidani A, et al Twist promotes tumor cell growth through YB-1 expression. Cancer Res. 2008;68(1):98–105. 10.1158/0008-5472.CAN-07-2981 18172301

[15] Franco HL, Casasnovas J, Rodríguez-Medina JR, Cadilla CL. Redundant or separate entities?—Roles of Twist1 and Twist2 as molecular switches during gene transcription. Nucleic Acids Res. 2011;39(4):1177–86. 10.1093/nar/gkq890 20935057 PMC3045590

[16] Connerney J, Andreeva V, Leshem Y, Mercado M a, Yang X, Lindner V, et al Sutures and Promote Suture Closure. 2009;318(2):323–34. 10.1016/j.ydbio.2008.03.037 PMC2605972 18471809

[17] Firulli AB, Conway SJ. Phosphoregulation of Twist1 provides a mechanism of cell fate control. Curr Med Chem. 2008;15(25):2641–7. 18855684 10.2174/092986708785908987 PMC2744367

[18] Connerney J, Andreeva V, Leshem Y, Muentener C, Mercado MA, Spicer DB. Twist1 dimer selection regulates cranial suture patterning and fusion. Dev Dyn. 2006;235(5):1345–57. 16502419 10.1002/dvdy.20717

[19] Bialek P, Kern B, Yang X, Schrock M, Sosic D, Hong N, et al A twist code determines the onset of osteoblast differentiation. Dev Cell. 2004;6(3):423–35. 15030764 10.1016/s1534-5807(04)00058-9

[20] Lee YS, Lee HH, Park J, Yoo EJ, Glackin C a., Choi Y Il, et al Twist2, a novel ADD1/SREBP1c interacting protein, represses the transcriptional activity of ADD1/SREBP1c. Nucleic Acids Res. 2003;31(24):7165–74. 14654692 10.1093/nar/gkg934 PMC291873

[21] Spicer D, Rhee J, Cheung W, Lassar A. Inhibition of myogenic bHLH and MEF2 transcription factors by the bHLH twist. Science. 1996;272(5267):1476–80. 8633239 10.1126/science.272.5267.1476

[22] Hamamori Y, Sartorelli V, Ogryzko V, Puri PL, Wu HY, Wang JYJ, et al Regulation of histone acetyltransferases p300 and PCAF by the bHLH protein twist and adenoviral oncoprotein E1A. Cell. 1999;96(3):405–13. 10025406 10.1016/s0092-8674(00)80553-x

[23] Gong XQ, Li. Dermo-1, a multifunctional basic helix-loop-helix protein, represses MyoD transactivation via the HLH domain, MEF2 interaction, and chromatin deacetylation. J Biol Chem. 2002;277(14):12310–7. 11809751 10.1074/jbc.M110228200

[24] Hamamori Y, Wu HY, Sartorelli V, Kedes L. The basic domain of myogenic basic helix-loop-helix (bHLH) proteins is the novel target for direct inhibition by another bHLH protein, Twist. Mol Cell Biol. 1997;17(11):6563–73. 9343420 10.1128/mcb.17.11.6563 PMC232510

[25] Piccinin S, Tonin E, Sessa S, Demontis S, Rossi S, Pecciarini L, et al A “Twist box” Code of p53 Inactivation: Twist box:p53 Interaction Promotes p53 Degradation. Cancer Cell. 2012;22(3):404–15. 10.1016/j.ccr.2012.08.003 22975381

[26] Pan D, Fujimoto M, Lopes A, Wang Y-X. Twist-1 Is a PPARδ-Inducible, Negative-Feedback Regulator of PGC-1α in Brown Fat Metabolism. Cell. 2009;137(1):73–86. 10.1016/j.cell.2009.01.051 19345188 PMC2688451

[27] Pettersson AT, Laurencikiene J, Mejhert N, Na E, Bouloumie A, Dahlman I, et al A Possible Inflammatory Role of Twist1 in Human White Adipocytes. Diabetes. 2010;59:564–671. 10.2337/db09-0997 20007935 PMC2828644

[28] Gitelman I. Evolution of the vertebrate Twist family and Synfunctionalization: A mechanism for differential gene loss through merging of expression domains. Mol Biol Evol. 2007;24(9):1912–25. 17567594 10.1093/molbev/msm120

[29] Germanguz I, Lev D, Waisman T, Kim CH, Gitelman I. Four twist genes in zebrafish, four expression patterns. Dev Dyn. 2007;236(9):2615–26. 17685477 10.1002/dvdy.21267

[30] Altschul SF, Gish W, Miller W, Myers EW, Lipman DJ. Basic local alignment search tool. J Mol Biol. 1990;215(3):403–10. 2231712 10.1016/S0022-2836(05)80360-2

[31] Sigrist CJ, De Castro E, Cerutti L, Cuche BA, Hulo N, Bridge A, et al New and continuing developments at PROSITE. Nucleic Acids Res. 2013;41(Database issue):D344–7. 10.1093/nar/gks1067 23161676 PMC3531220

[32] Singh S, Gramolini AO. Characterization of sequences in human TWIST required for nuclear localization. BMC Cell Biol. 2009;10:47 10.1186/1471-2121-10-47 19534813 PMC2709654

[33] Lichtarge O, Bourne HR, Cohen FE. An Evolutionary Trace Method Defines Binding Surfaces Common to Protein Families. J Mol Biol [Internet]. 1996 3 [cited 2016 Jun 15];257(2):342–58. Available from: http://linkinghub.elsevier.com/retrieve/pii/S0022283696901679 8609628 10.1006/jmbi.1996.0167

[34] Innis CA, Shi J, Blundell TL. Evolutionary trace analysis of TGF- and related growth factors: implications for site-directed mutagenesis. Protein Eng Des Sel. 2000;13(12):839–47. 10.1093/protein/13.12.839 11239083

[35] Kinexus Bioinformatics Corporation: Protein-Ser/Thr Kinase Consensus Phosphorylation Site Specificity [Internet]. [cited 2015 Jun 5]. p. 1. Available from: http://www.kinexus.ca/pdf/graphs_charts/ProteinSerKinaseSpecificity.pdf

[36] Wright PE, Dyson HJ. Intrinsically unstructured proteins: re-assessing the protein structure-function paradigm. J Mol Biol. 1999;293(2):321–31. 10550212 10.1006/jmbi.1999.3110

[37] Dunker a. K, Brown CJ, Lawson JD, Iakoucheva LM, Obradović Z. Intrinsic disorder and protein function. Biochemistry. 2002;41(21):6573–82. 12022860 10.1021/bi012159+

[38] Uversky VN. Natively unfolded proteins: a point where biology waits for physics. Protein Sci. 2002;11(4):739–56. 11910019 10.1110/ps.4210102 PMC2373528

[39] Dunker a. K, Lawson JD, Brown CJ, Williams RM, Romero P, Oh JS, et al Intrinsically disordered protein. J Mol Graph Model. 2001;19(1):26–59. 11381529 10.1016/s1093-3263(00)00138-8

[40] Maia AM, da SIlva JH, Mencalha AL, Caffarena ER, Abdelhay E. Computational modeling of the bHLH domain of the transcription factor TWIST1 and R118C, S144R and K145E mutants. BMC Bioinformatics. 2012;13(1):184. 22839202 10.1186/1471-2105-13-184 PMC3507644

[41] Buchan DWA, Minneci F, Nugent TCO, Bryson K, Jones DT. Scalable web services for the PSIPRED Protein Analysis Workbench. Nucleic Acids Res. 2013;41(W1): W349–W357. 23748958 10.1093/nar/gkt381 PMC3692098

[42] Kelley LA, Sternberg MJE. Protein structure prediction on the Web: a case study using the Phyre server. Nat Protoc. 2009;4(3):363–71. 10.1038/nprot.2009.2 19247286

[43] Varadi M, Guharoy M, Zsolyomi F, Tompa P. DisCons: a novel tool to quantify and classify evolutionary conservation of intrinsic protein disorder. BMC Bioinformatics. 2015;16:153 10.1186/s12859-015-0592-2 25968230 PMC4427981

[44] Rogelj B, Godin K, Shaw C, Ule J. the Functions of Glycine-Rich Regions in Tdp-43, Fus and Related Rna-Binding Proteins In: Zdravko L, editor. RNA Binding Proteins [Internet]. Landes Bioscience and Springer Science; 2011 p. 1–17. Available from: http://www.landesbioscience.com/pdf/03Lorkovic_Ule_.pdf

[45] Rumpold H, Rhodes GH, Bloch PL, Carson D a, Vaughan JH, Pines NT. The Epstein-Barr Nuclear Antigen-1 (EBNA-1) l. J Immunol. 1987;138(2):593–9. 2432130

[46] Moitra K, Scally M, McGee K, Lancaster G, Gold B, Dean M. Molecular evolutionary analysis of ABCB5: the ancestral gene is a full transporter with potentially deleterious single nucleotide polymorphisms. PLoS One. 2011;6(1):e16318 Available from: 10.1371/journal.pone.0016318 10.1371/journal.pone.0016318 21298007 PMC3029322

[47] Pelé J, Bécu J-M, Abdi H, Chabbert M. Bios2mds: an R package for comparing orthologous protein families by metric multidimensional scaling. BMC Bioinformatics. 2012;13(1):133. 22702410 10.1186/1471-2105-13-133 PMC3403911

[48] Hillis DM, Heath TA, St John K. Analysis and visualization of tree space. Syst Biol. 2005;54(3):471–82. 16012112 10.1080/10635150590946961

[49] Kendall M and Coijin C. Mapping phylogenetic trees to reveal distinct patterns of evolution. 2015. bioRXiv. Available at: http://biorxiv.org/content/biorxiv/early/2015/09/11/026641.full.pdf 10.1093/molbev/msw124 PMC5026250 27343287

[50] Zhong J, Ogura K, Wang Z, Inuzuka H. Degradation of the transcription factor Twist, an oncoprotein that promotes cancer metastasis. Discov Med. 2013;15(80):7–15. 23375009 PMC5522964

[51] Varadi M, Vranken W, Guharoy M, Tompa P. Computational approaches for inferring the functions of intrinsically disordered proteins. Front Mol Biosci. 2015;2(8):1–8. 26301226 10.3389/fmolb.2015.00045 PMC4525029

[52] Babu MM, van der Lee R, de Groot NS, Gsponer J. Intrinsically disordered proteins: Regulation and disease. Curr Opin Struct Biol. 2011;21(3):432–40. 10.1016/j.sbi.2011.03.011 21514144

[53] Bocca SN, Magioli C, Mangeon A, Junqueira RM, Cardeal V, Margis R, et al Survey of glycine-rich proteins (GRPs) in the Eucalyptus expressed sequence tag database (ForEST). Genet Mol Biol. 2005;28(3 SUPPL.):608–24.

[54] Di Tommaso P, Moretti S, Xenarios I, Orobitg M, Montanyola A, Chang JM, et al T-Coffee: A web server for the multiple sequence alignment of protein and RNA sequences using structural information and homology extension. Nucleic Acids Res. 2011;39(suppl 2):W13–7. 21558174 10.1093/nar/gkr245 PMC3125728

[55] Kemena C, Notredame C. Upcoming challenges for multiple sequence alignment methods in the high-throughput era. Bioinformatics. 2009;25(19):2455–65. 10.1093/bioinformatics/btp452 19648142 PMC2752613

[56] Procter JB, Thompson J, Letunic I, Creevey C, Jossinet F, Barton GJ. Visualization of multiple alignments, phylogenies and gene family evolution. Nat Methods. 2010;7(3 Suppl):S16–25. 10.1038/nmeth.1434 20195253

[57] Talavera G, Castresana J. Improvement of phylogenies after removing divergent and ambiguously aligned blocks from protein sequence alignments. Syst Biol. 2007;56(4):564–77. 17654362 10.1080/10635150701472164

[58] Schrödinger L. The PyMOL Molecular Graphics System, Version 1.7.4 Schrödinger, LLC. [Internet]. Available from: https://www.pymol.org/

[59] Capella-Gutiérrez S, Silla-Martínez JM, Gabaldón T. trimAl: A tool for automated alignment trimming in large-scale phylogenetic analyses. Bioinformatics. 2009;25(15):1972–3. 10.1093/bioinformatics/btp348 19505945 PMC2712344

[60] Ropelewski AJ, Nicholas HB, Mendez RRG. MPI-PHYLIP: Parallelizing computationally intensive phylogenetic analysis routines for the analysis of large protein families. PLoS One. 2010;5(11):e13999 10.1371/journal.pone.0013999 21085574 PMC2981553

[61] Rambaut A. Fig Tree from A. Rambaut [Internet]. 2007. Available from: http://tree.bio.ed.ac.uk/software/figtree/

