# Correction: Evolution of the Twist Subfamily Vertebrate Proteins: Discovery of a Signature Motif and Origin of the Twist1 Glycine-Rich Motifs in the Amino-Terminus Disordered Domain

**DOI:** 10.1371/journal.pone.0164848

**Published:** 2016-10-12

**Authors:** Yacidzohara Rodriguez, Ricardo R. Gonzalez-Mendez, Carmen L. Cadilla

The images for Figs 8 and 9 are incorrectly switched. The image that appears as Fig 8 should be Fig 9, and the image that appears as Fig 9 should be Fig 8. The figure captions appear in the correct order.

**Fig 8:**
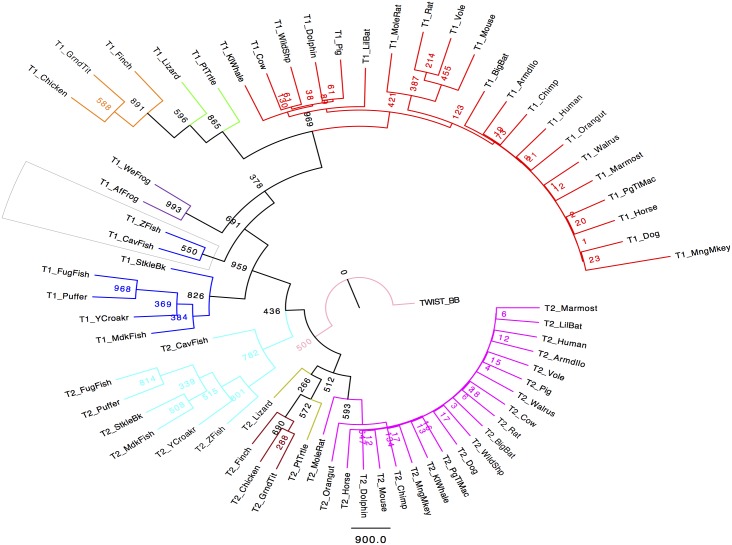
Phylogenetic analysis of vertebrate Twist proteins. The outgroup Twist protein (Twist_BB) is located at the root of the tree. The analysis shows that the Twist vertebrate family underwent a gene duplication event that split Twist proteins into two main clades: Twist1 and Twist2. Different vertebrate species are represented by the following colors: Magenta (Twist2 mammals), Yellow (Twist2 reptiles), Brown (Twist2 birds), Cyan blue (Twist2 fish), Blue (Twist1 fish), Purple (Twist1 amphibians), Green (Twist1 reptiles), Orange (Twist1 birds), Red (Twist1 mammals). Numbers on each node display bootstrap values. The TrimAl package in the gappyout mode was used to trim the alignment. A maximum likelihood tree was constructed using PHYLIP (MPIproml) with 1000 bootstrap replicates. The program FigTree was used to visualize the phylogenetic tree. Sequence names used represent the common name of the species to which they belong.

**Fig 9:**
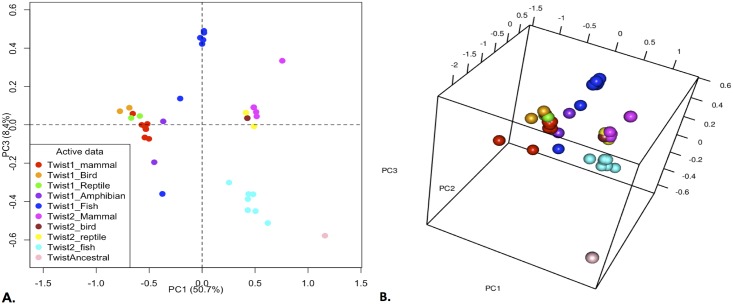
Largest Principal Components of the MMDS Analysis for the Twist1 and Twist2 proteins. A) Two-dimensional plot of principal components 1 and 3 of the MMDS analysis. B) Three-dimensional plot of the most important principal components determined from the MMDS analysis. It can be seen that the Twist2 are closer to the Twist_BB in sequence space. Also, that the Twist1 sequences show more evolutionary drift over sequence space. Different vertebrate species are represented by the following colors: Magenta (Twist2 mammals), Yellow (Twist2 reptiles), Brown (Twist2 birds), Cyan blue (Twist2 fish), Blue (Twist1 fish), Purple (Twist1 amphibians), Green (Twist1 reptiles), Orange (Twist1 birds), Red (Twist1 mammals).

## References

[1] Rodriguez Y, Gonzalez-Mendez RR, Cadilla CL (2016) Evolution of the Twist Subfamily Vertebrate Proteins: Discovery of a Signature Motif and Origin of the Twist1 Glycine-Rich Motifs in the Amino-Terminus Disordered Domain. PLoS ONE 11(8): e0161029 doi: 10.1371/journal.pone.0161029 27556926 10.1371/journal.pone.0161029 PMC4996418

