# Supplementary figures and images for: Evolution of the Twist Subfamily Vertebrate Proteins: Discovery of a Signature Motif and Origin of the Twist1 Glycine-Rich Motifs in the Amino-Terminus Disordered Domain
Source: PLoS One. 2016 Aug 24;11(8):e0161029. doi: 10.1371/journal.pone.0161029 (PMC4996418; doi:10.1371/journal.pone.0161029)

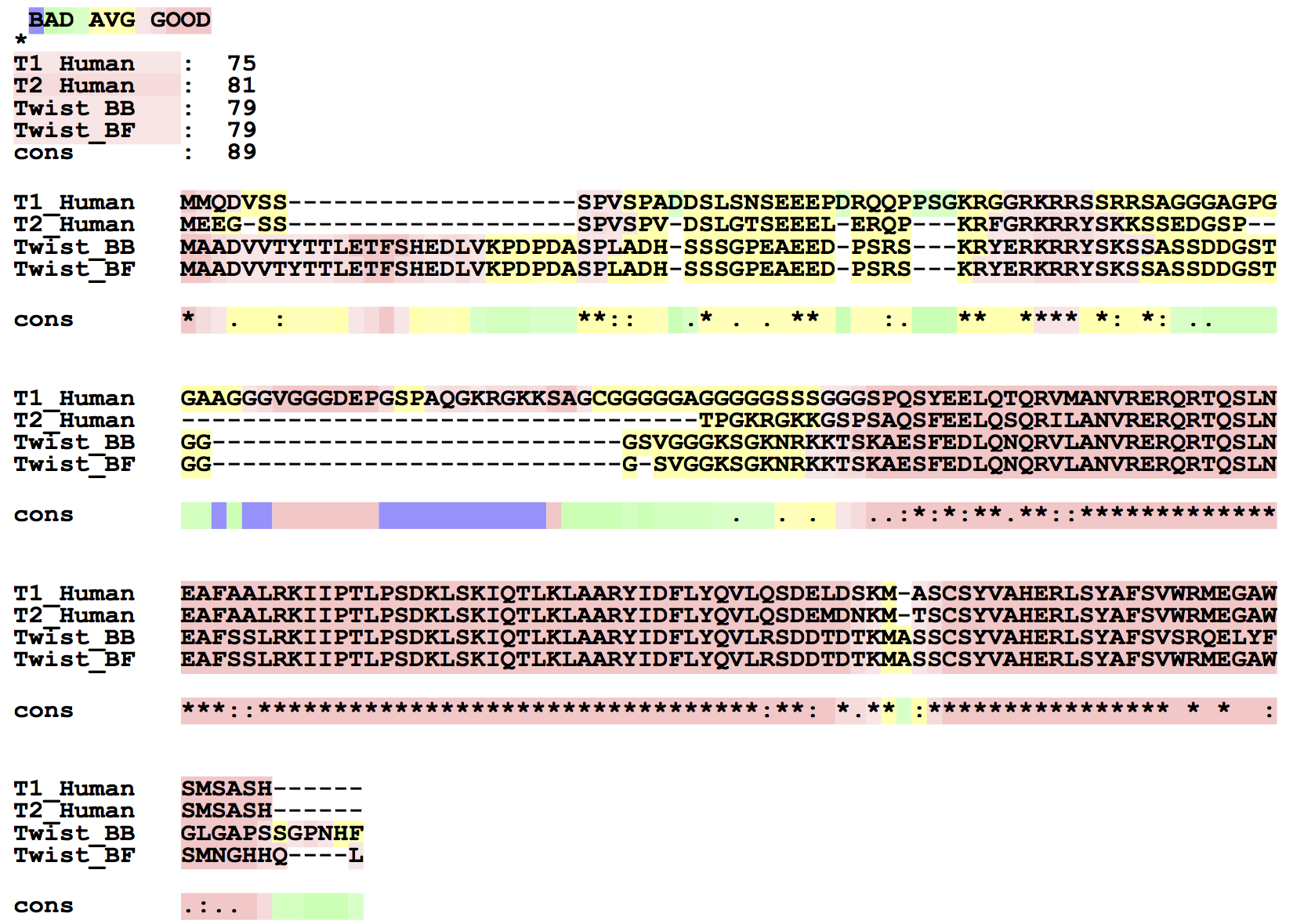

Supplement: S1 Fig — Branchiostoma belcheri (BB) and Branchiostoma floridae (BF); both share approximately 89% bHLH identity when compared to human Twist sequences. Both ancestor proteins contain approximately three or four glycine surrounding the second glycine-rich region, which further suggest this region evolved first. Overall, when compared with BF, Twist2 shares 68% amino acid identify while Twist1 shares 56%, which further suggests that the ancestor of both Twist paralogs was a “Twist2-like” protein. Below the protein sequence: (*) = Good conserved residues (dark pink); (:) = average conservative mutations (yellow); (.) = semi-conservative mutations (pink); () = non-conservative mutations (purple and green are indicative of badly conserved residues). Twist_BB = Branchiostoma belcheri species. Twist_BF = Branchiostoma floridae. The alignment was performed with PSI-COFFEE. (TIFF) [file pone.0161029.s001.tiff]

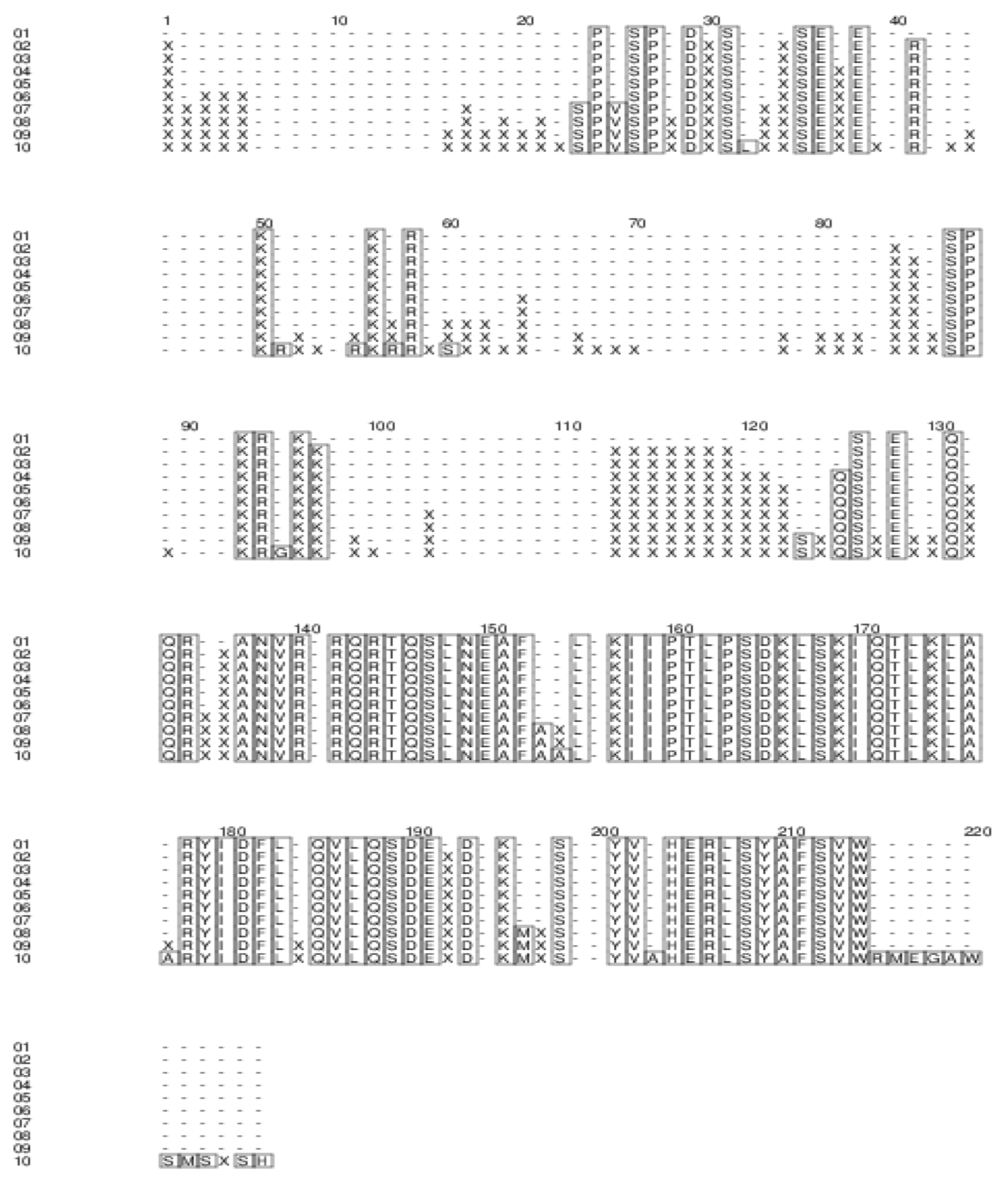

Supplement: S2 Fig — Conserved residues are surrounded by boxes, while class-specific residues, particularly the as Asparagine (for Twist1) and Threonine (for Twist2) are denoted by an X. The ET results also detected the same sequence motif found using PROSITE. (TIFF) [file pone.0161029.s002.tiff]
